# A map of intra- and intercellular immune responses across diverse in vitro stimuli and inflammatory disease

**DOI:** 10.1186/s13073-026-01721-y

**Published:** 2026-07-15

**Authors:** Oliver Wood, Adam T. Braithwaite, Josephine Fisher, Li Li, Lynne Murray, Christopher Paluch, Matthew A. Jackson-Wood

**Affiliations:** 1 Gilead Sciences, 9400 Oxford Business Park, Garsington, Oxford, OX4 2HN UK; 2 Gilead Sciences, 333 Lakeside Dr, Foster City, CA 94404 USA

**Keywords:** Immunology, Single-cell RNA-seq, Inflammatory disease, Model systems

## Abstract

**Background:**

In vitro stimulation of healthy human immune cells is widely used to model the immune states observed in disease, both to investigate pathology and to test therapeutic approaches. However, experiments typically focus on individual cell types or stimuli and a comprehensive cellular comparison of common immunomodulators and their relevance to disease is lacking.

**Methods:**

We used single-cell transcriptomics to generate a reference profile of human peripheral blood mononuclear cells treated with 11 different in vitro stimuli, totalling over 150,000 cells across 21 immune cell types. We demonstrate its utility by performing comparative analyses across the immunomodulatory conditions and against peripheral blood profiles from patients with inflammatory disease.

**Results:**

We describe transcriptomic responses both unique to and shared across stimuli. For instance, stimulation via the T cell receptor (anti-CD3, CytoStim™) and IFN-α induced broad activation signatures, including indirect effects across multiple cell types, whereas TNF-α and LPS elicited more restricted, cell-specific responses. Ligand-receptor interaction mapping also uncovered the dominant intercellular signalling pathways in each stimulation. Comparing to patient datasets, we identified several aspects of inflammatory disease recapitulated by stimuli. For example, IFN-α stimulation induced SLE-like signatures across cell types, whereas LPS did so specifically within monocytes. However, comparative cell–cell network analysis showed that in vitro stimuli were only able to recapitulate some, but not all, aspects of intercellular interactions upregulated in SLE, highlighting the limitations of these model systems.

**Conclusions:**

This dataset provides a valuable resource for understanding the effects of common in vitro blood stimuli, offering insights into their similarities and differences at cellular resolution, and, as demonstrated here, helping to guide the appropriate use of in vitro systems to model disease.

**Supplementary Information:**

The online version contains supplementary material available at https://doi.org/10.1186/s13073-026-01721-y.

## Background

Profiling immune cells and their responses to perturbations is central to research in disease contexts such as autoimmunity and cancer. However, access to patient tissues presents both logistical and scale-related challenges: samples are often difficult to obtain, and substantial inter-patient heterogeneity necessitates large cohorts for adequate statistical power. To address this, researchers routinely use immune cell activation assays with peripheral blood mononuclear cells (PBMCs), often from healthy donors, as a more accessible and controlled primary cell system to elucidate inflammatory mechanisms and for pre-clinical testing of potential therapeutics using a range of molecular readouts [1]. Examples include stimulating immune cells with interferons to reproduce the biology of systemic lupus erythematosus (SLE) [2], with toll-like receptor agonists such as lipopolysaccharide (LPS) as a model of bacterial or viral infection [3], or using TNF-α stimulation to represent rheumatoid arthritis (RA) biology [4]. A standardised, comparable profile of cellular transcriptomic responses to common in vitro stimuli would present a valuable resource, providing a detailed understanding of the biological pathways that are shared and distinct across them whilst also highlighting their strengths and limitations as models of disease. Previous studies profiling transcriptional responses to ex vivo stimuli have had narrow scope in terms of diversity of cells and/or stimulation conditions. For instance, they have used isolated cell types rather than whole PBMCs, e.g. dendritic cells [5], neutrophils [6], monocytes/macrophages [7–9] or T cells [9–11]; or they have profiled multiple cell types but with a single or limited number of conditions, e.g. LPS [12] or anti-CD3/CD28 + IL-2 [13, 14]. The largest studies have extended to comparison of only three stimuli [15, 16]. A more recent study profiled responses across a range of cytokines head-to-head but focused on immune cells from mouse lymph nodes after 4 hours of in vivo stimulation [17]. Thus, a comprehensive, comparative, high-resolution map of immune responses across human cell type populations and commonly used in vitro stimuli is lacking.

To address this gap, we applied single-cell RNA sequencing (scRNA-seq) to profile the responses of healthy donor PBMCs to 11 different immunomodulatory stimuli. These were selected to include common stimuli, both broadly activating and inhibitory, targeting diverse pathways and cell types. Our primary aim was to generate a comparative reference resource for effects of commonly used in vitro immune stimuli and to illustrate how such a resource can be used to assess disease relevance, regulatory programmes, and intercellular communication. Integration of the data across donors and conditions enabled systematic comparisons of the effects of each condition within 21 identified immune cell subtypes, including elucidation of differential cell–cell communication pathways in the mixed-cell system. As an example of the utility of this resource, we compared the stimulation data to transcriptomic data from immune cells sorted from the blood of patients with a range of immune-mediated diseases [18, 19] and identified the in vitro stimuli most representative of each indication. Furthermore, focusing on single-cell data from SLE patients, we identified specific cell–cell interactions enriched in disease and how they are (and are not) recapitulated by different stimuli. These results provide a high-resolution reference for the transcriptional effects of commonly used immunomodulatory conditions at the cellular level. They establish a foundational resource for understanding immune cell dynamics, designing immune activation studies, and exploring the molecular mechanisms underlying immune-mediated disease.

## Methods

### Sample collection and in vitro stimuli

Leukocyte blood cones from healthy, anonymized donors were obtained from the NHSBT Oxford Blood Donor Centre under REC reference 21/PR/0798, protocol number Miro_HT01, and IRAS project ID 300191. PBMCs were isolated by density-gradient centrifugation. Briefly, diluted blood cone cells were layered over Ficoll-Paque PLUS Media (Cytiva) in a 50 mL Leucosep tube (Greiner Bio-One GmbH). The resulting cells were seeded into Nunc™ Non-Treated 24-well plates (Thermo Fisher Scientific) at a density of 8 × 10^5^/cm^2^ in a final volume of 1 mL RPMI (Gibco), supplemented with Penicillin–Streptomycin-Neomycin (Gibco), 10% Foetal Bovine Serum (Gibco) and L-Glutamine (Gibco). A total of 12 separate donors were used, with stimulations carried out across 2 phases, with each batch including 6 different donors and a baseline (unstimulated) condition. The stimulation conditions used were 1 ng/mL Ultra-LEAF Purified anti-human CD3 antibody, 10 ng/mL human TGF-β1, 1 ng/mL GMP Human IL-1β, 10 ng/mL human IL-4 GMP, 10 ng/mL human TNF-α GMP, 10 ng/mL human IL-2 GMP, 10 ng/mL human IL-10 GMP, 5 ng/mL human IFN-α (Biolegend), 100 ng/mL lipopolysaccharide (LPS) solution (eBiosciences), 20 µl/mL CytoStim™ human (Miltenyi Biotec), and the B cell stimulator cells (8:1, PBMC:BCS) with 100 ng/mL IL-21 (Biolegend). Pilot experiments were performed to establish an optimal timepoint and concentrations for stimulation (as assessed using common markers by flow cytometry) whilst minimising changes to cell populations (Additional File 1: Fig. S1 and Fig. S2). B cell stimulation required 48 h to achieve sufficient activation; however, longer incubation led to a decline in myeloid cell populations. Accordingly, 48 h was selected as the experimental time point, and concentrations for the other stimuli were optimised for this duration. Concentrations for CytoStim™ and IFN-α were selected based on the manufacturer’s recommendations and prior experimental experience, respectively.

The B cell stimulator cells consisted of HEK293 (ATCC) cells transduced to express CD40L and an anti-CD79B ScFv construct. These were seeded at a density of 1 × 10^5^/cm^2^ on the day prior to the experiment and were grown in Dulbecco's Modified Eagle Medium supplemented with Penicillin–Streptomycin-Neomycin (Gibco), 10% Foetal Bovine Serum (Gibco) and L-Glutamine (Gibco).

### Flow cytometric evaluation

Following in vitro stimulation, 2 × 10^5^ of the harvested cells were used to determine the cell proportions and activation readouts using fluorescence-activated cell sorting (FACS) on a BD FACSAria™ Fusion. The activation and cell composition readouts were captured with cell surface staining using antibodies from Biolegend® for CD38 (HIT2), CD25 (MA251), HLA-DR (L243), CD86 (FUN-1) and PD-1 (EH12.2H7). Surface staining was carried out for 30 min at 4 °C after an initial Fc receptor block step using Human TruStain FcX™ (Biolegend ®) according to the manufacturer’s instructions, in sorting buffer containing PBS, 2 mM EDTA, and 2% Foetal Bovine Serum. The cell markers for lineage determination and cell proportion analysis were CD8 (SK1), CD14 (M5E2), CD19 (HIB19), CD56 (5.1H11) CD3 (HIT3a) and CD4 (OKT4). Live cells were gated using DAPI (4',6-diamino-2-phenylindole, dihydrochloride) staining solution (Miltenyi Biotec).

### Immune cell sorting and bulk RNA-seq

Bulk RNA-seq was performed to assess how well the single-cell RNA-seq data, which have lower transcriptomic coverage, captured transcriptomic shifts observed with deeper sequencing. For a subset of the stimuli (BCS, CytoStim™, LPS, and IFN-α) and baseline (unstimulated) conditions, following the 48-h in vitro assay, the major cell populations were processed for FACS on the BD FACSAria™ Fusion using the staining method described above. Between 3 × 10^5^ and 1 × 10^6^ cells were sorted into sorting buffer using core lineage markers for monocytes (CD14^+^); CD4^+^ T cells (CD3^+^, CD4^+^); CD8^+^ T cells (CD3^+^, CD8^+^) and B cells (CD19^+^ and CD20^+^). RNA was isolated using the RNeasy mini kit (Qiagen) and libraries were prepared using the NEBNext rRNA Depletion Kit and NEBNext Ultra II Directional RNA Library Prep Kit for Illumina according to the manufacturer’s protocol. The prepared libraries were then sequenced on an Illumina NovaSeq 6000 S4 in 150 bp paired end read mode to achieve at least 20 million reads per sample. Reads corresponding to residual ribosomal RNA were removed using SortMeRNA [20]. The RNA-seq reads were processed to gene counts using the nf-core rnaseq pipeline (v3.12) [21] using Salmon [22] for pseudoalignment to the Ensembl GRCh38.96 reference genome. Outputs were imported to R using *tximport* [23], and differential expression analysis between baseline and stimulation calculated using DESeq2 [24], including donor as a covariate. Samples that clustered separately from all other samples in principal component analysis (PCA) were considered outliers and excluded from differential expression testing; however, all samples, including those excluded from analysis, were retained in the GEO deposition and annotated accordingly. Similarities to the DEGs identified in single-cell analyses were assessed by Pearson correlation of the bulk log2 fold change values with those from equivalent cell populations in scRNA-seq profiling, considering all genes significant in either analysis.

### Single-cell library preparation and sequencing

Harvested cells from the in vitro stimulation were fixed using the Evercode™ cell fixation kit v2 (Parse Biosciences) according to the manufacturer’s protocol. The average yield of fixed cells taken forward to SPLiT-seq (split-pool ligation-based transcriptome sequencing) was 5 × 10^5^. Single-cell RNA-seq library preparation and sequencing were performed using the Evercode™ WT 100 k Whole Transcriptome Kit v2 (Parse Biosciences) according to the manufacturer’s protocol. The initial fixed cell samples were counted using a Countess 3 (Thermo Fisher Scientific) cell counter to achieve 100,000 cells per sample-tag barcode. Cells from multiple biological samples were combinatorially barcoded and pooled during library preparation. After barcoding, eight sub-libraries were prepared, each containing ~ 10,000 cells. The final libraries underwent standard library QC and quantification followed by sequencing on two NovaSeq X Plus (Illumina) 25b 300 cycle Flow Cell Lanes, set to 150 bp paired end read mode. Raw FASTQ files from pooled sub-libraries were processed using the Parse pipeline (v1.2.1) for demultiplexing, trimming, filtering, and alignment to the GRCh38 reference genome; assignment of reads to cell barcodes; per-sample QC and filtering of cells versus ambient RNA; and output of filtered count matrices for downstream analyses.

### Single-cell transcriptomic profiles

Single-cell transcriptomic profiles were processed using Seurat (v5.1.0) [25] in R. Raw counts were normalised and log-transformed; variable features were identified using the variance-stabilising transformation method. Low complexity or low-quality cells were removed as defined by > 15% mitochondrial UMIs or < = 0.8 log10-genes per UMI. Genes with zero detected counts across all cells were removed. Preliminary, per-cell automated cell type prediction was performed with SingleR (v2.4.1) [26] using a sorted PBMC bulk RNA-seq reference dataset [27]. Doublets were called separately for each donor–stimulation sample using Doublet Finder (v2.0.4) [28], with sample-specific principal component selection and pK optimisation. An expected doublet rate of 3% was adjusted for homotypic doublets based on preliminary SingleR annotations, and predicted doublets were removed before downstream analysis. An initial UMAP dimensional reduction and clustering were performed (resolution = 0.50, first 30 principal components based on 3000 variable genes), which enabled the removal of the remaining B cell stimulator cells identified by clustering separately from PBMC populations and expression of non-PBMC markers. Cell cycle and batch effects on final cell clustering and annotation were minimised by integrating across samples with reciprocal PCA [29]. Using baseline samples as a reference, integration was performed using the first 30 principal components, based on a consensus set of 4000 variable genes identified across samples by the variance-stabilising transformation method, with cell cycle genes excluded and variable genes scaled while regressing out total RNA count and percentage mitochondrial UMIs. Post-integration UMAP dimensional reduction and clustering were performed (resolution = 1.4) for visualisation and to assign lineage labels (T/ILC, B, myeloid) (Additional File 1: Fig. S3a), then cells were re-clustered separately within each lineage at multiple resolutions (0.2, 0.4, 0.6, 0.8, 1.0). Within each lineage, the expression patterns of relevant marker genes were assessed per cluster to inform selection of final resolutions per lineage (T/ILC, 0.4; B, 0.2; myeloid, 0.4) and assign cluster IDs based on the chosen clustering. Differentially expressed genes were determined per cluster versus all other clusters using a Wilcoxon rank-sum test, and cell types were annotated based on the top marker genes per cluster (Additional File 1: Fig. S3b). Finally, low-quality, suspected doublet and mixed clusters based on mixed lineage marker expression were removed from downstream analyses. Downstream differential expression analyses comparing cell types or stimulation conditions were performed on pseudo-bulk profiles as described below, rather than on single-cell expression values.

### Assessment of cell type- and condition-specific effects

Differential cell type compositional analysis was performed using Propeller [30], comparing each condition with baseline in a mixed-effects model with donor included as a random effect. Aggregated pseudo-bulk transcriptomic profiles were generated per cell type (separately at the general and subtype level as appropriate), condition, and donor. Pseudo-bulk profiles were used to calculate differentially expressed genes per cell type (either versus all other cells in the baseline condition, for cell type-specific genes; or for each condition vs. baseline, for stimulation-responsive genes) with DESeq2 (v1.42.1) [24] while adjusting for donor effects. DEGs were defined by Benjamini–Hochberg adjusted *p* < 0.05 unless a different stringency threshold or additional criteria (such as log2-fold change) were required for specific analyses (and are stated as such). Pseudo-bulk profiles were used to reduce the effect of drop-out associated with the low transcriptomic coverage of scRNA-seq data, and comparisons to bulk RNA-seq from sorted cell populations confirmed that the single-cell results reflect those from a deeper sequencing approach (Additional File 1: Fig. S3c).

Comparisons of the altered transcriptomic profiles within and between datasets were summarised by the following metrics, where appropriate: Jaccard index of DEGs, defined as the ratio of the intersection size to the union size; correlation of log2-fold changes, using Pearson correlation unless otherwise stated; and normalised enrichment score (NES) from gene set enrichment analysis of gene positions within gene profiles ranked by Wald statistic (log2-fold change divided by standard error of log2-fold change) with the fgsea package (v1.28.0) [31]. Functional annotation of DEG profiles was also performed by over-representation analysis of DEGs (adjusted *p* < 0.05) compared to the background universe of all detected genes using fgsea with Reactome pathway annotations of size 30–300 [32]. MultiNicheNet [33] (v2.0.1) was used to assess cell–cell communication, comparing cells from each condition to baseline, with donor as a covariate and the following options: min_sample_prop = 0.50; fraction_cutoff = 0.05; empirical_pval = FALSE; logFC_threshold = 1.5; p_val_threshold = 0.05; p_val_adj = FALSE; top_n_target = 250. Interaction edge lists produced by MultiNicheNet were converted to networks for each stimulation, selecting the top 2000 edges based on the MultiNicheNet prioritisation score. This threshold was selected by generating networks with increasing numbers of edges and comparing the overlap between conditions to identify a network size with enough edges for similarities and differences between conditions to be observed, but not so many that the networks became homogenous (Additional File 1: Fig. S4).

### Transcription factor activity inference

TF activity was inferred per cell using decoupleR [34] with univariate linear modelling using the CollecTRI TF-target annotations [35]. Mean TF activity was calculated per donor-minor cell type-stimulation to create pseudo-bulk profiles for differential testing between cell types or conditions and visualisation. Differential TF activity was calculated from these profiles per cell type at baseline, and for stimulation versus baseline in each cell type, using limma [36] and adjusting for donor effects. Significant differential TF activity was defined by globally FDR-adjusted *p* < 0.05. The donor-adjusted delta TF activity scores were used for filtering and plotting relative activity across cell types and stimulation conditions. The signed TF-target gene annotations were used to annotate TFs by their predominant effect on target genes: activation, > 80% positive; repression, > 80% negative; mixed, all others. Delta TF activity scores were compared by Pearson correlation with log2-fold changes for corresponding TF RNA from the pseudo-bulk comparisons (stimulation versus baseline), across all TFs whose activity was significantly altered in any cell type or stimulation combination compared to baseline.

### Additional datasets

Gene count matrices from sorted bulk RNA-seq profiles from the ImmunexUT study [19, 37] were obtained from the National Bioscience Database Center (NBDC) Human Database. Cell type-specific DEG profiles (Benjamini–Hochberg adjusted *p* < 0.01, log2-fold change > 0.5) for each disease vs. healthy were generated using limma [36]. scRNA-seq profiles from SLE and normal cells [18, 38] were obtained as a pre-processed Seurat object from BioTuring [39]. The Seurat anchor-finding and label transfer functionality was used to guide reannotation of the SLE/normal cells using labels from the in vitro stimulated PBMCs, with some manual adjustment of cell annotations where appropriate. DEGs were assessed per cell type for SLE vs. normal cells with the pseudo-bulk DESeq2 method as previously used. Ligand-receptor interactions were assessed with the MultiNicheNet method as previously used, but without adjustment of donor as a covariate (as groups were mutually exclusive in this dataset). scRNA-seq profiles from mice treated with cytokines [17, 40] were obtained as pre-processed Seurat objects from the authors. DEGs were assessed per cell type as cells from treated vs. untreated mice with the Wilcoxon rank sum test (adjusted *p* < 0.05). Mouse gene symbols were converted to human gene symbols based on one-to-one orthologs as determined via NCBI HomoloGene [41] and biomaRt [42]. Differential gene profiles for human PBMC stimuli vs. baseline were generated for cells with matching annotation levels (B cell, NK cell, CD4^+^ T cell, CD8^+^ T cell, regulatory T cell, cDC1, cDC2 and pDC) to the available mouse data using an analogous cell-level Wilcoxon rank-sum test (adjusted *p* < 0.05) to enable comparison between datasets.

## Results

### Changes in cellular composition across different in vitro stimuli

**Fig. 1:**
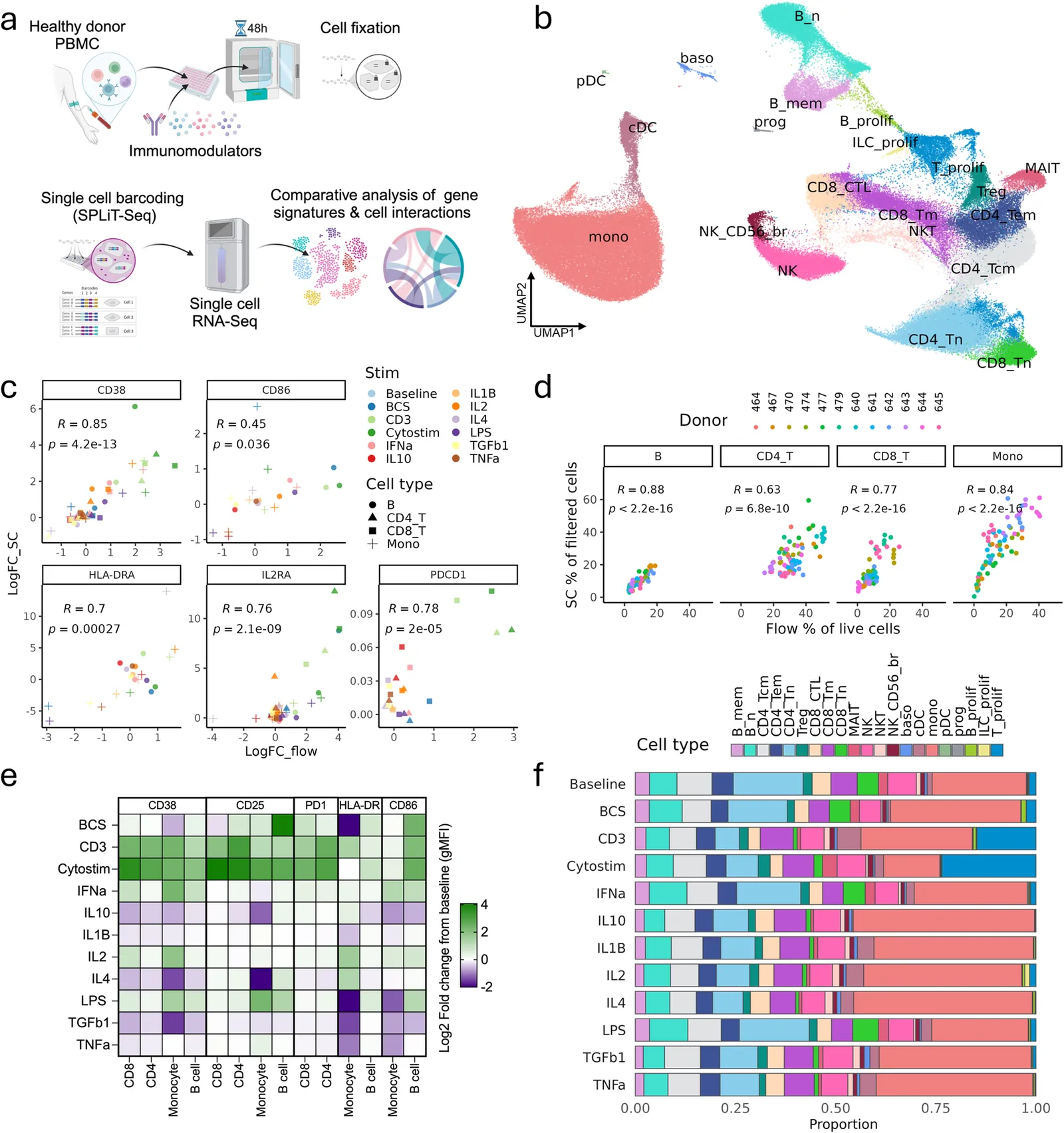
Creating a map of responses to 11 immunomodulators across 21 immune cell types. **A** Schematic overview of the experimental and computational workflow. **B** UMAP dimensional reduction of 153,443 cells integrated across conditions. **C** Pearson correlation of log2-fold changes (gMFI) from flow cytometry protein measurements vs. log2-fold change normalised counts from scRNA-seq data (stimulation vs. baseline) for activation markers. For scRNA-seq data, profiles were summarised as mean pseudo-bulk profiles per general cell type/stimulation to calculate fold changes vs. baseline. **D** Spearman correlation of broad cell type (B cell, CD4, CD8 and monocytes) frequencies between flow cytometry and scRNA-seq, split by cell type across donors. **E** Altered levels of surface protein activation markers determined by flow cytometry in CD4^+^ T cells, CD8^+^ T cells, B cells and monocytes. Mean log2-fold change (gMFI) is shown for each condition vs. baseline. **F** Combined cell proportions from 6 donors per stimulation and 12 donors (2 batches of 6) for baseline. Cell types of > = 1% are labelled. B_mem, memory B cells; B_n, naive B cells; CD4_Tcm, central memory CD4 T cells; CD4_Tem, effector memory CD4 T cells; CD4_Tn, naive CD4 T cells; Treg, T regulatory cells; CD8_CTL, CD8 cytotoxic T cells; CD8_Tm, memory CD8 T cells; CD8_Tn, naive CD8 T cells; MAIT, mucosal-associated invariant T cells; NK, CD56-dim natural killer cells; NK_CD56_br, CD56-bright NK cells; NKT, NK T cells; baso, basophils; cDC, conventional dendritic cells; mono, monocytes; pDC, plasmacytoid dendritic cells; prog, progenitor cells; B_prolif, proliferating B cells; ILC_prolif, proliferating ILC; T_prolif, proliferating T cells

We treated PBMCs from healthy human donors with anti-CD3 antibody, TGF-β1, IL-1β, IL-4, TNF-α, IL-2, IL-10, IFN-α, LPS, CytoStim™, or a B cell stimulator cell line (BCS) expressing CD40L and an anti-CD79B construct (Fig. 1a). The experiment was performed by splitting the stimulation conditions into two batches with 6 donors profiled in each (for a total of 12 unique individuals). After 48 h, baseline (untreated) and post-treatment samples were profiled by scRNA-seq. The 48-h timepoint and stimuli concentrations were optimised to ensure sufficient stimulation, as measured using conventional flow cytometry markers, whilst maintaining representation of all major cell populations (Additional File 1: Fig. S1 and S2). We identified 21 immune cell subtypes across 153,443 cells (Fig. 1b). Condition- and donor-specific clustering effects were removed for the purposes of annotation, based on canonical marker expression (Additional File 1: Fig. S3a-b).

Before detailed analyses of the scRNA-seq profiles, we validated them against cell marker expression (Fig. 1c) and cell type frequencies (Fig. 1d) assessed using flow cytometry; and bulk RNA-seq profiles from sorted cells. High-level immune cell frequencies determined by flow cytometric profiling were significantly correlated with equivalent cell types from scRNA-seq profiles (Fig. 1d). Anti-CD3 and CytoStim™ treatment induced upregulation of established flow cytometry activation markers, particularly in CD4^+^ and CD8^+^ T cells (Fig. 1e). Inhibitory conditions such as IL-4, IL-10, and TGF-β1 reduced activation marker expression (e.g., CD38, CD25) in monocytes, whereas 48-h LPS treatment led to a tolerance response, with reduced expression of the antigen-presentation-associated markers HLA-DR and CD86, as previously described [43]. These protein expression measurements were significantly positively correlated with their cognate mRNAs in the scRNA-seq data (Fig. 1c). Discrepancies between technologies were only noted for PD-1 (*PDCD1*), which exhibited low RNA detection, reflecting previous observations [44]. Overall, the flow cytometry comparisons showed good alignment of activation marker and cell type composition with scRNA-seq data. In a subset of cells/stimuli also profiled by bulk RNA-seq and the bulk-derived differential gene expression results were highly correlated to those from scRNA-seq data; showing that the single-cell transcriptomic profiles were representative of changes observed when using deeper sequencing approaches (Additional File 1: Fig. S3c).

Analysis of cell type composition in the scRNA-seq profiles showed monocytes were the most abundant cell type, representing ~ 30% of cells (Additional File 1: Fig. S3d). Using more finely grained cell type annotations, we observed overall differences in post-treatment cellular composition across stimuli (Fig. 1f) and inter-donor variability (Additional File 1: Fig. S3e). Applying compositional statistical analysis, CytoStim™ and anti-CD3 conditions had the highest number of significant proportional shifts, with 6 cell types altered in each (Additional File 1: Fig. S3f). The profiles resulting from these treatments were largely similar, with lower naïve *CD4*^+^ and *CD8*^+^ T cells and a corresponding higher relative abundance of proliferating T cells (driven to the greatest extent by CytoStim™). A relative increase in proliferating T cells was also driven by the IL-2 and TGF-β1 treatments.

Myeloid proportions were affected by a range of conditions, particularly conventional dendritic cells (cDCs), which were increased in abundance with CytoStim™, anti-CD3, IFN-α and LPS treatment but lower in IL-10. Plasmacytoid dendritic cell (pDC) relative abundance was lower with CytoStim™, LPS and BCS. The BCS stimulation was the only condition in which monocytes were significantly more abundant. BCS also caused the greatest relative increase in proliferating B cells as expected. Notably, two conditions (TNF-α and IL-1β) did not induce any significant changes in cell type composition.

An important consideration is that these are compositional data generated after filtering out low‑quality cells, including damaged or dying cells, and reflect relative abundances of viable cells at the time of capture relative to the other cell types. To identify compositional changes resulting from stimulation driven proliferation, we assessed cell cycle phase across the high-level cell type annotations (Additional File 1: Fig. S3g). As expected, signatures of cell cycle progression were increased in T cells under the T cell-targeted stimuli (anti-CD3 and CytoStim™), and to a lesser extent with BCS, whereas B cells were strongly shifted to S phase by the BCS condition. A similar shift to S phase was also induced by IL-2 in the ILCs, while IL-4 elicited a shift from G2M to G1 in B cells. Notably, although we observed an increased relative abundance of the proliferating B cell cluster in the anti-CD3 condition (Fig. 1f), the absence of corresponding cell cycle shift in B cells (Additional File 1: Fig. S3g) suggests that this likely reflects changes in other cell populations rather than active B cell proliferation. Collectively, these data indicate that stimuli produce distinct compositional changes in PBMCs over 48 h, shaping the biology captured in each condition.

### Cell types display distinct transcriptional responses to stimuli

**Fig. 2:**
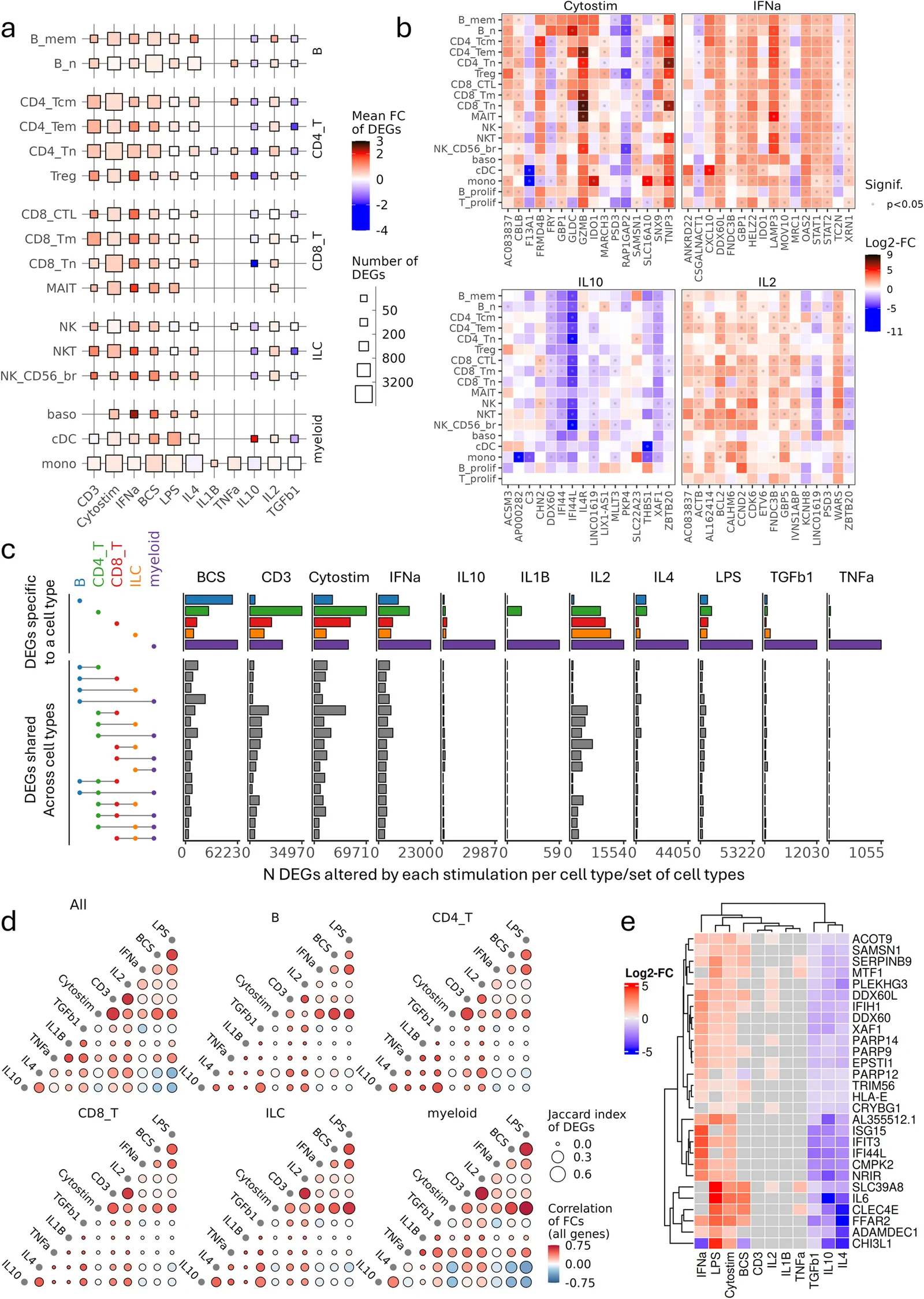
Quantitative comparison of the overall transcriptomic responses. **A** Transcriptomic changes within pseudo-bulk profiles per cell type comparing each condition to baseline. Number of differentially expressed genes (DEGs; adjusted *p* < 0.05) and mean log2-fold change (FC) of all DEGs is shown per comparison. **B** Log2-fold changes for top 4 DEGs per lineage (B, T, ILC, myeloid) by lowest p-values, for key stimulations vs. baseline. **C** Specific and shared DEGs per stimulation vs. baseline across general cell types. For overlapping DEG sets, those with > = 2000 shared genes in at least 2 general cell types are shown. **D** Similarity of DEG profiles between conditions evaluated by Pearson correlation of FC for all genes and Jaccard similarity index of DEG sets. Comparisons were based on pseudo-bulk DEG profiles from all cells or general cell types. **E** Log2-FC for significant DEGs (adjusted *p* < 0.05) upregulated by CytoStim™ and downregulated by IL-4, IL-10 and TGF-β1, but not altered by anti-CD3, in myeloid lineage cells

Next, we evaluated the magnitude of transcriptional responses elicited by each stimulation on each cell type (available in Additional File 2: Table S1). Anti-CD3 and CytoStim™ induced widespread transcriptional activation in T cells as expected but also in B and myeloid cells (Fig. 2a). The CytoStim™ treatment, which binds both T cell receptor and MHC molecules on antigen-presenting cells, elicited a larger effect in monocytes and cDCs compared to anti-CD3, which engages the T cell receptor of T cells and Fc receptors of myeloid populations. Conversely, inhibitory conditions such as TGF-β1 and IL-10 reduced gene expression in T cells and monocytes. However, IL-10 uniquely upregulated a small subset of genes in cDCs, while TGF-β1 increased gene expression in mucosal-associated invariant T (MAIT) cells and regulatory T cells (Tregs).

Examining the most altered genes in each cell type-stimulation combination, we observed both cell type-specific and independent changes (Fig. 2b and Additional File 1: Fig. S5a). For example, IFN-α increased expression of *LAMP3* broadly across all cell types, whereas *CXCL10* was most elevated in cDCs, central memory CD4^+^ T cells and memory B cells. CytoStim™ increased *IDO1* in B cells and monocytes, whereas *SLC16A10* was upregulated only in monocytes. The top genes altered with IL-10 had reduced expression across cell types and included a decrease in the interferon signature genes *IFI44L* and *IFI44*. The expression of *BCL2*, which blocks programmed cell death [45], was most strongly upregulated in CD8 + T and CD56-dim NK cells in response to IL-2.

To explore broader transcriptional changes, we compared differentially expressed gene (DEG) sets across cell types within each condition (Fig. 2c). This revealed that, for a given stimulation, cell type-specific DEGs were more numerous than those shared between cell types. This was particularly apparent in myeloid cells, which had a high number of unique DEGs in almost all conditions. However, some responses were shared across cell types – for example, myeloid and B cells stimulated with BCS shared overlapping DEGs. Thus, whilst there were a small number of stimulation-induced transcriptional responses that were conserved across cell types, such as the IFN gene signature (Fig. 2b), the wider transcriptional signature was largely unique to cell types within a stimulus.

Performing the opposite contrast – assessing DEG signatures across different stimuli (Fig. 2d) – highlighted similarities in transcriptional responses. Across all cells, positive correlations were observed between IFN-α, LPS and CytoStim™, which in turn were negatively correlated with the inhibitory conditions TGF-β1, IL-4, and IL-10. This pattern was broadly recapitulated within individual cell types; with some notable distinctions. For example, in myeloid cells, anti-CD3 stimulation showed strong similarity to CytoStim™ but lacked the inverse correlations with TGF-β1, IL-4, and IL-10. To explore this further, we identified a set of genes upregulated by CytoStim™, but not by anti-CD3, in myeloid cells, that were also downregulated by IL-4, IL-10 and TGF-β1 (Fig. 2e). This set included interferon-signature genes (e.g. *ISG15, IFIT3, IFI44L*) and the cytokine *IL-6*. These differences may reflect direct engagement of MHC-bearing myeloid cells by CytoStim™, a component absent from anti-CD3 stimulation.

To explore the functional effects of stimuli, we clustered variable genes to identify shared and distinct pathways across cell type-stimulation combinations (Additional File 1: Fig. S5b). IFN-α stimulation increased interferon pathway expression broadly across cell types, and this was also seen in T cells and ILCs stimulated with anti-CD3 and CytoStim™. A reduction in these activation pathways was observed with IL-4 and IL-10 treatment in myeloid cells, and with TGF-β1, IL-10 and IL-4 treatment in ILCs and T cells. BCS elevated cell cycle/proliferation pathways in B cells, an effect also observed in T cells treated with CytoStim™ or anti-CD3. Overall, our results demonstrate that stimuli elicit both shared and distinct transcriptional programmes, but that the dominant responses remain strongly cell type dependent, motivating a closer examination of the regulatory factors underlying these patterns.

### Identification of stimulation-specific transcriptional regulation

**Fig. 3:**
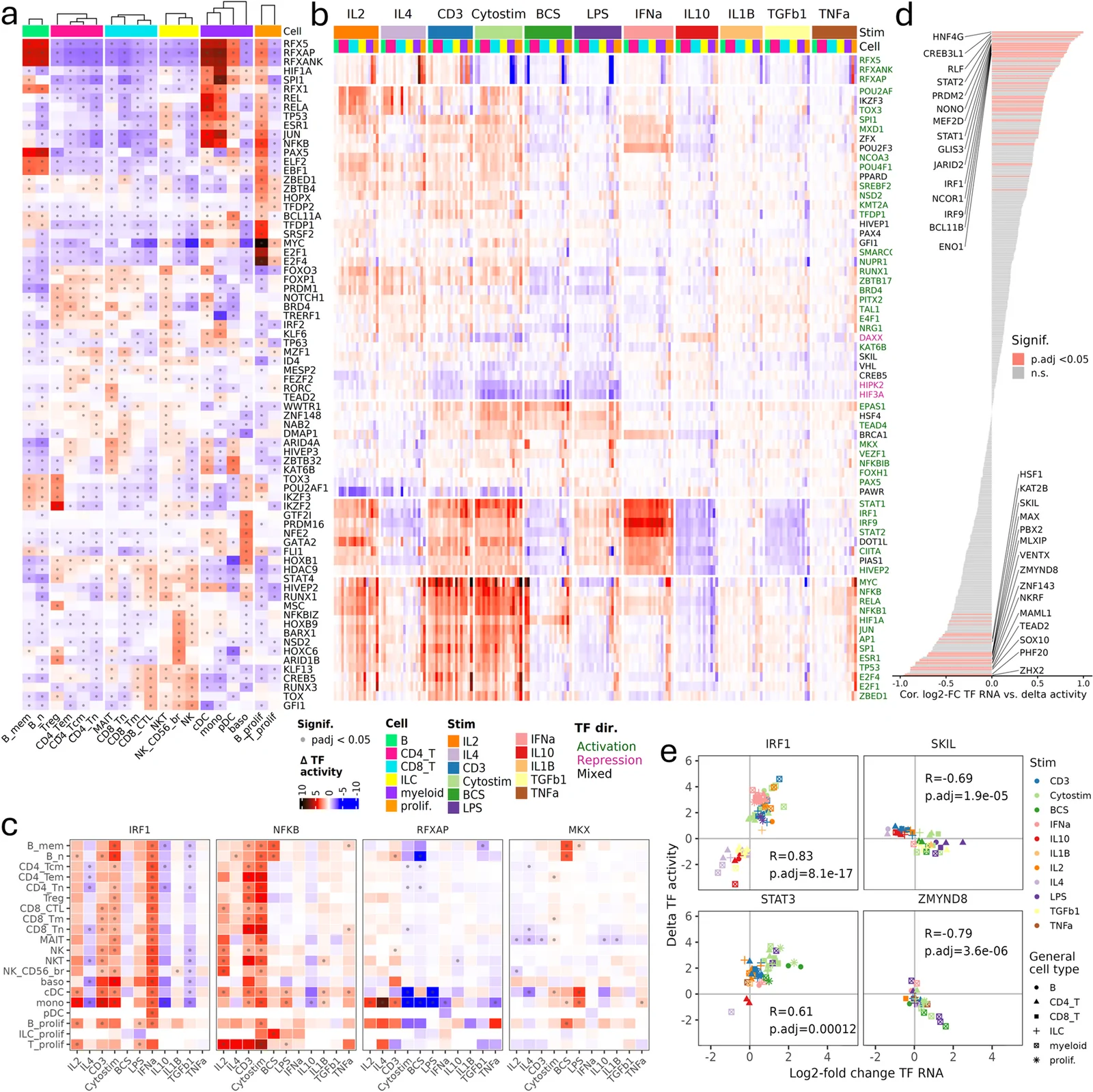
Transcriptional regulation in response to immunomodulation. TF activity was inferred per cell with decoupleR univariate linear modelling and CollecTRI TF-target annotations; activity profiles were averaged per donor/cell type/condition. **A** Mean baseline differential TF activity per minor cell type (vs. mean other cell types) for the top 6 significant TFs (adjusted *p* < 0.05) by delta activity, requiring positive mean activity within the cell type. Colour bars indicate general cell types. **B** Differential TF activity for stimulation vs. baseline in each cell type. Columns represent a minor cell type–stimulation combinations, with colour bar annotation of general cell type and stimulation. The top 3 significant TFs (adjusted *p* < 0.05) by absolute delta activity per general cell type-stimulation combination are shown. TFs (rows) are grouped by k-means clustering (k = 5). TF names are coloured by predominant signed effect on target genes: Activation, > 80% positive; Repression, > 80% negative; Mixed, all others. **C** Delta TF activity (stimulation vs. baseline) for selected representative TFs from 4 of the groups, across cell types and conditions. **D** Pearson correlation R of log2-fold change, TF RNA vs. delta TF activity across minor cell type-stimulations vs. baseline. Data used for correlations were any cell type-condition combination with significant (adjusted *p* < 0.05) altered activity. Each bar represents a TF; significant (adjusted *p* < 0.05) are coloured red; top 15 significant positive and negatively correlated TFs are labelled. **E** Key TFs from the top correlated across minor cell type-stimulations where TF activity was significantly altered vs. baseline (adjusted *p* < 0.05). Colour denotes stimulation and shape, general cell type

We inferred transcription factor (TF) activity from the expression of CollecTRI [35]-annotated TF–target genes to identify drivers associated with the observed transcriptional patterns at baseline and in response to stimulation (Additional File 3: Table S2). To validate this approach, we compared cell types at baseline and observed expected lineage- and cell type-specific patterns of TF activity (Fig. 3a), including PAX5 in B cells [46], IKZF2 (Helios) in regulatory T cells [47], GATA2 in basophils [48] and BCL11A in pDCs [49]. FOXP3 was also highly enriched in regulatory T cells, albeit below the cut-off used to define the top TFs visualised. We then applied this method to score TF activity across all cells and stimuli and identified TFs whose activity was most consistently altered across stimulation-cell type combinations, which highlighted expected major regulators of inflammation (Additional File 1: Fig. S5c). These included the type I interferon pathway TFs (IRF1, IRF9, STAT1 and STAT2), the NFκB pathway, including TFs encoded by NFKB1, NFKB2, REL, RELA (p65) and RELB (p52), and SIM2, NAB2, and HIF3A, which consistently had reduced activity across stimuli and cell types.

We then considered the top TFs active in each cell type-stimulus combination individually revealing both global stimulation effects and cell type-specific responses (Fig. 3b). At a high level, inferred TF activity largely separated stimulatory from inhibitory conditions: with the aforementioned canonical inflammatory regulators such as NFKB1 and RELA showing higher activity across IL-2-, anti-CD3- and CytoStim™-treated populations, whereas inhibitory conditions such as IL-10 and TGF-β1 were associated with lower activity. Interferon-associated TFs including STAT1, IRF1, and IRF9, were most active across all cell types with IFN-α, as expected, but were also active across broad cell types in the other activating conditions. More specific patterns included MKX that was selectively active in specific cell types in the LPS and BCS conditions, Regulatory Factor X TFs that were most active in proliferating cells stimulated by IL-2 and IL-4, and higher activity of DAXX specific to IL-10 stimulation. The latter was also one of the only dominantly repressive TFs amongst the top active TFs, whereas most others were classified as predominantly gene-activating TFs.

Representative TFs with distinct activity patterns at broad cell type resolution were further examined across cell subsets (Fig. 3c). IRF1 had a wide activation-associated pattern, with strongest activity under IFN-α but also elevated activity under CytoStim™, anti-CD3 and IL-2, while IL-4, IL-10 and TGF-β1 reduced its activity. NFKB similarly marked a broad activating programme, with strongest activity under CytoStim™ and anti-CD3, especially in T/ILC populations, and with additional myeloid-specific induction in several contexts (LPS, IL-1β and TNF-α). Unlike IRF1, however, NFKB was not strongly induced by IFN‑α. RFXAP and MKX showed more cell type-selective patterns. RFXAP, a core component of the RFX complex required for MHC class II transcription [50], showed selective enrichment in IL‑4‑, anti‑CD3‑ and IL‑2‑treated settings in myeloid cells, and with anti‑CD3 in naïve B cells. Conversely, RFXAP activity was strongly reduced in myeloid cells by LPS, CytoStim™ and BCS, and in naïve B cells under BCS, indicating context‑dependent modulation of antigen‑presentation pathways. MKX showed strong induction in LPS‑treated myeloid cells, consistent with its reported association with myeloid activation [51].

Comparing changes in TF RNA abundance with changes in downstream TF activity across cell type-stimulation combinations (Fig. 3d), we found that only a minority of TFs had a significant positive correlation between TF activity and TF abundance. Representative examples include *IRF1* and *STAT3*, which showed strong positive correlations, and *ZMYND8* and *SKIL*, which showed significant negative correlations between TF RNA abundance and inferred TF activity (Fig. 3d-e). This highlights the value of transcriptome-wide data for TF activity inference in capturing the complexity of the multiple regulatory layers linking TF transcription to functional activity. For example, in the case of SKIL, this inverse relationship may reflect a combination of a known negative feedback mechanism within TGF‑β signalling [52] and regulon overlap with other TFs, including SMAD4 (9 shared targets in CollecTRI; representing 43% of SKIL targets), which could contribute to activity being inferred from shared target genes. Overall, these analyses highlight the utility of this resource for exploring the complex regulatory mechanisms underlying transcriptomic responses to immune stimuli.

### Differences in cell–cell communication networks between stimuli

**Fig. 4:**
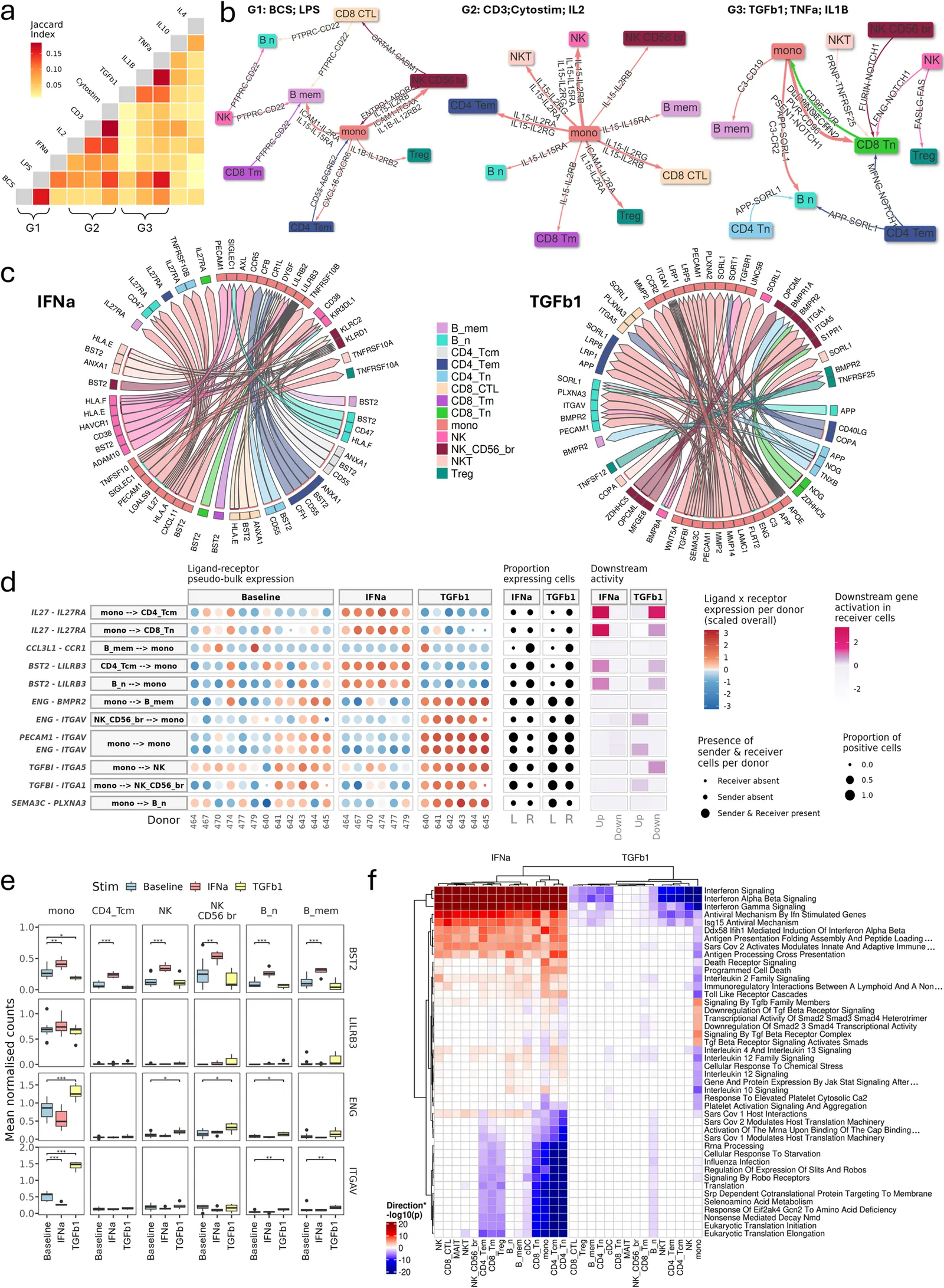
Differential ligand-receptor interactions driven by stimulation conditions. Upregulated ligand-receptor interactions were predicted per condition compared to baseline with MultiNicheNet. **A** Overall similarity of the intercellular networks per condition (defined by the top 2000 upregulated interactions) compared by Jaccard indices. Three groups of conditions were defined by relatively higher similarity in their signaling networks (BCS and LPS; IL-2, CytoStim™ and anti-CD3; TGF-β1, TNF-α and IL-1β) and in (**B**) the top 15 upregulated interactions are shown for each group. Edge colour represents the predicted sender cell type and edge width indicates the number of ligand-receptor interactions for the cell type pair. **C** Summary of the top 50 upregulated interactions for the IFN-α and TGF-β1 conditions. **D** Highlighted supporting data for key ligand-receptor interactions in the IFN-α and TGF-β1 conditions across donors. Data shown per ligand-receptor and sender-receiver cell (1 per row), from left to right are: scaled ligand-receptor pseudo-bulk expression product and sender/receiver cell presence per donor/condition; fraction of cells expressing ligand/receptor; downstream ligand activity (up- or down-regulated target genes) in receiver cells. **E** Expression of key ligands and receptors in specific cell types and stimulation conditions. Data are summarised with each point representing mean value per cell type/donor/stim. **F** Enriched Reactome pathways in each cell type, for IFN-α and TGF-β1 conditions compared to baseline. Enrichment derived from over-representation of genes that were up- or down-regulated (adjusted *p* < 0.05), with colour representing direction multiplied by the significance (inverse log10 p-value). The top 40 pathways by mean absolute deviation of plotted values across all cell types and conditions are shown

We next explored how different stimuli influenced intercellular communication between cell types. Upregulated ligand-receptor interactions between cell types were predicted for each condition, and the resulting networks were compared across stimuli (Fig. 4a, Additional File 4: Table S3). Overall overlap between the differential interaction networks was low (Jaccard < 0.2 in all comparisons), indicating limited sharing of individual interactions across conditions. Nevertheless, 8 of the 11 stimuli showed relative similarities that allowed them to be grouped into three broad signalling clusters: BCS and LPS (group 1); IL-2, anti-CD3 and CytoStim™ (group 2); and IL-1β, TNF-α and TGF-β1 (group 3). Among the top 15 interactions within each group, we found that a third of those in Group 1 involved signalling into B cells via CD22 from CD8^+^ T cells and CD56-dim NK cells, Group 2 was dominated by IL-15-associated signalling from monocytes, and Group 3 contained more interactions involving naïve CD8^+^ T cells (Fig. 4b).

Exploring the interaction network of each stimulation individually, we also identified stimulation-specific cell type interactions (Additional File 1: Fig. S6a). For instance, these included NOG-BMPR2 binding between CD4^+^ T cells and CD56-bright NK cells stimulated with TGF-β1, and IL27-IL27RA interactions between IFN-α-stimulated T cells and monocytes. Examination of the top 50 upregulated ligand-receptor interactions per condition (Additional File 1: Fig. S6b) showed that the cell–cell interaction network for CytoStim™ stimulation included well-known binding partners between T cells and antigen-presenting cells (B cells, monocytes and DCs), such as CD80 and CD274 on monocytes interacting with CTLA4 and PDCD1 on T cells. Treatment with CytoStim™ also increased FASLG on CD56-bright and CD56-dim NK cells and FAS expression on regulatory T cells, as well as IL-15 expression by B cells interacting with T cells.

We provide a more detailed comparison of these networks for the IFN-α and TGF-β1 conditions, given their generally opposing effects and diverse networks (Fig. 4c and Additional File 1: Fig. S6c-d), and show the expression data for receptors, ligands, and their downstream pathway genes at the donor level for unique interactions (Fig. 4d). Overall, at the donor level there was higher baseline variation between individuals than in the post-treatment conditions, suggesting biological convergence under in vitro stimulation. Monocyte *IL27* and T cell *IL27RA* expression were elevated in the IFN-α condition and reduced with TGF-β1, which was reflected in downstream target gene expression in T cells. One of the most prominent cell–cell interactions in IFN-α-stimulated cells was BST2 in T cells and B cells interacting with LILRB3 on monocytes. The distinct expression profiles of *BST2* and *LILRB3* at the mRNA level (Fig. 4e) highlighted that *LILRB3* remained constitutively expressed in monocytes, but *BST2* was upregulated in central memory CD4^+^ T cells, CD56-bright and CD56-dim NK cells, and B cells with IFN-α treatment. Key signalling induced by TGF-β1 included: increased *ENG* in monocytes, which communicates via BMPR2 on B cells and ITGAV on NK cells and monocytes (auto-regulatory), and the production of TGF-β1 by monocytes that can interact with integrins ITGA5 and ITGA1 expressed on NK cells [53]. These intercellular pathways were associated with expected intracellular transcriptional differences when comparing IFN-α and TGF-β1 directly, with IFN-α stimulating interferon pathways that were suppressed by TGF-β1 and clear activation of TGF-β pathways with TGF-β1 (Fig. 4f, Additional File 5: Table S4). These findings highlight cell–cell communication networks as potential targets for interrupting activation cascades within mixed-cell populations and demonstrate the utility of these data for exploring intercellular dynamics.

### Identifying aspects of disease biology reproduced by in vitro stimuli

**Fig. 5:**
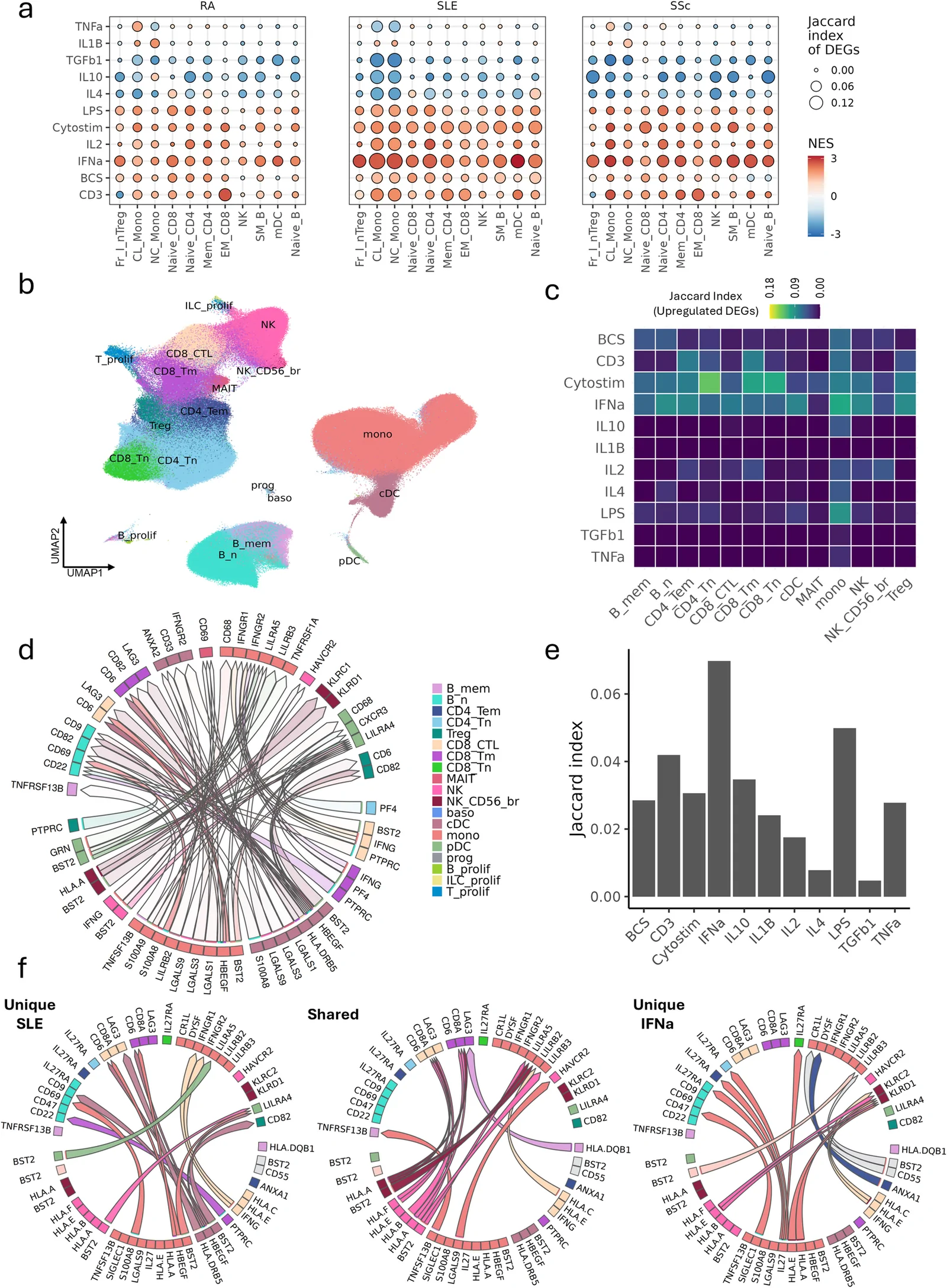
Cell type-specific signatures of disease within immunomodulatory conditions. **A** Gene set enrichment of cell type-specific disease gene sets (defined as adjusted *p* < 0.01, log2-fold change > 0.5; disease vs. healthy) from bulk sorted PBMCs (ImmunexUT dataset—rheumatoid arthritis, RA; systemic lupus erythematosus, SLE; systemic sclerosis, SSc) within ranked gene profiles (ranked by Wald stat; logFC/lfcSE; stimulation vs. baseline) of matching cell types of stimulated PBMC profiles. Enrichment is summarised as normalised enrichment score (NES) and overlap of differentially expressed genes (DEGs) between disease and stimulation (defined as adjusted *p* < 0.01, log2-fold change > 0.5; stimulated vs. baseline) by Jaccard index. **B** UMAP dimensional reduction of SLE and normal single PBMCs with mapped cell type labels. Overall proportions for cell types with n > = 100 are shown. **C** Jaccard indices of per-cell type upregulated DEGs (adjusted *p* < 0.05) from SLE vs. normal cells, compared to upregulated DEGs from each cell type/stimulation vs. baseline. **D** Summary of the top 50 upregulated interactions in SLE vs. normal. **E** Jaccard indices of the top 2000 upregulated interactions from SLE compared to those from each stimulation condition. **F** Shared and unique upregulated interactions within the top 2000 for SLE vs. normal, and IFN-α vs baseline. The top 15 interactions from unique SLE, shared and unique IFN-α are shown. Up to 3 interactions are shown per cell type pair

As an example of the translational utility of the stimulation data, we compared their transcriptomic signatures and cell type interaction networks with those derived from patients with immune-mediated disease to assess which aspects of disease biology were captured by each stimulus. First, we compared cell type-specific stimulation signatures with those derived from bulk RNA-seq of sorted PBMC populations from 10 inflammatory diseases in the ImmuNexUT cohort [19] (Additional File 1: Fig. S7a-b). Absolute overlap in DEG sets was modest, which is not unexpected given differences in gene coverage between scRNA-seq and bulk RNA-seq, together with the use of stringent adjusted p-value and fold change cutoffs to define DEG sets in both data sets. To provide a more quantitative relative comparison of representation between stimuli, we assessed the enrichment of ImmuNexUT disease signatures across the ranked single-cell stimulation profiles. Overall, genes upregulated in disease were positively enriched in activating stimuli (IL-2, BCS, anti-CD3, LPS, IFN-α, and CytoStim™) and negatively enriched in immunosuppressive conditions (TGF-β1, IL-10, IL-4 and TNF-α). Several diseases were relatively poorly represented across most stimuli, including ANCA-associated vasculitis and Takayasu arteritis. Certain stimuli were also more highly enriched for signatures from specific diseases. For example, focusing on RA, SLE and systemic sclerosis (SSc) (Fig. 5a), IFN-α-treated cells showed enrichment of genes associated with all three conditions, whereas CytoStim™ and LPS showed greater overlaps with SLE than with RA or SSc. Notably, most stimuli showed consistent patterns of enrichment across disease signatures (either positive, weak, or negative) across all cell types.

To demonstrate the utility of our stimulation data for comparative analyses at cellular resolution, we focused on a scRNA-seq dataset from patients with SLE, a disease with well-established peripheral inflammatory signatures that showed overlap with several in vitro stimuli in the prior bulk RNA-seq analyses. We reannotated published single-cell RNA-seq data from PBMCs of SLE patients and healthy donors to facilitate direct comparison to our in *vitro* stimuli (Fig. 5b, Additional File 1: Fig. S7c-d) [18]. SLE signatures were most closely reproduced by CytoStim™ and IFN-α stimuli (relative to the other stimuli) across the majority of cell types, but with distinct patterns between the two (Fig. 5c). Other conditions show more limited, cell type-specific upregulation of SLE-associated genes, such as in LPS stimulation of monocytes. Interestingly, while MAIT cells were extensively transcriptionally altered in SLE (n DEGs = 3,781; Additional File 1:Fig. S7e), consistent with previous observations in circulating MAIT cells from SLE patients [54], this was not reproduced by any in vitro stimulation. In contrast, monocytes showed SLE-like transcriptional changes under multiple stimuli.

We next assessed the inter-cell type interactions enriched in SLE (Fig. 5d) and found that many of the dominant signalling pathways, such as BST2-LILRB3 between monocytes and T cells, were also enriched within the IFN-α stimulated PBMCs (Fig. 4c). Furthermore, when comparing the global cell type interaction network of SLE to each stimulation (Fig. 5e), IFN-α had the highest overlap to the SLE network. However, whilst the IFN-α and SLE networks shared many overlapping edges, unique interactions were also enriched in each (Fig. 5f). The SLE network uniquely included B cells interacting with monocytes (via CD69-S100A8). Conversely, IL-27 signalling was prevalent in the IFN-α stimulation network but not the SLE derived network. Overall, these comparative analyses reiterate the value of in vitro stimulation systems as models of human disease, but also the need to understand the limits of their representation.

As a final comparison with alternate model systems, we explored the similarity of our in vitro signatures to those from in vivo models recently described in the Immune Dictionary dataset [17] (Additional File 1: Fig. S7f-g). This study assessed effects of cytokines 4 h after administration in mice. The largest overlap between our in vitro data and in vivo effects (in terms of number of DEGs) was observed across all cell types after IFN-α treatment (Additional File 1: Fig. S7f). However, in general, the overall extent of transcriptional shifts in the murine cytokine treatments was much smaller than in our in vitro system (Additional File 1: Fig. S7g), and there were low and, in some cases, negative correlations between them. This is not unexpected given the species, tissular, and protocol differences between the data sets and reiterates the necessity to understand how representative model systems are of the desired biology.

## Discussion

This study provides a foundational resource for understanding immune responses to a range of commonly used in vitro stimuli, capturing both their direct and indirect effects within a mixed immune cell population and, as illustrated by our exemplar application to SLE, how closely they reproduce disease-relevant features. We found that although some stimuli produced convergent effects within specific cell types – for example, anti-CD3, CytoStim™ and IL-2 on T cells – there was relatively little overlap in the transcriptional changes induced by a given stimulus across different cell types. The magnitude and specificity of effects also varied substantially between conditions. For example, CytoStim™ and IFN-α induced large transcriptional changes across T cells and additional cell lineages, whereas anti-CD3 or TNF-α may be more appropriate when the aim is to restrict effects more narrowly to T cell populations. At the cell type level, monocytes showed some of the largest transcriptional shifts. The broader responsiveness of myeloid cells relative to T cells may reflect their intrinsic plasticity and heterogeneity, together with their broad functional repertoire in cytokine and chemokine sensing, antigen presentation, and the coordination of secondary immune responses [55]. Across stimuli, the greatest transcriptional divergence was, as expected, between the largely immunosuppressive effects of TGF-β1, IL-10, and, in monocytes, IL-4, and the activating conditions. Notably, however, the CD8^+^ T cell signatures from patients with immune disease showed positive correlation with IL-4-induced changes in CD8^+^ T cells, highlighting complexity beyond a simple activating-versus-inhibitory framework and underscoring the value of cellular-level reference data.

As a further layer of analysis, ligand-receptor interaction profiling revealed key mechanisms of inter-cell type communication. For example, IFN-α stimulation induced *IL27* production by monocytes, which was predicted to act on T cells via the IL27RA receptor. This is consistent with the role of IL-27 in autoimmune regulation, where T cells responding to IL-27 can increase the anti-inflammatory cytokine IL-10, inhibit T-helper 17 cell differentiation, and reduce IL-17 [56]. IL-27 has been implicated in inflammatory and autoimmune-related diseases, such as inflammatory bowel disease, where it is likely involved in an inadequate anti-inflammatory response [57]. TGF-β1 treatment modulated myeloid-B cell interactions through the upregulation of *ENG* and *BMPR2*, which can impact B cell maturation and long-term humoral immunity [58, 59]. Prior studies have demonstrated similar immunoregulatory effects of these cytokines, supporting alignment of the data resource with expected effects [60]. The ligand-receptor interaction analysis also identified more complex networks, such as the upregulation of *BST2* in IFN-α-stimulated T and B cells, which interacted with LILRB3 on monocytes. BST2 has been implicated in type I interferon signalling and autoimmune diseases such as SLE [18]. While these results are inherently limited by the quality and completeness of interaction databases, the analysis provides an example of how these data can reveal key signalling pathways within immune cell networks. Recent methodological developments are enabling profiling of intracellular proteins alongside surface receptors and transcriptomes at the single-cell level [61]; such approaches could enable direct measurement of signalling molecules and test the cell–cell signalling potential and directionality inferred from mRNA here.

The data also provide an opportunity to compare gene regulation within cell types and across stimuli. We explored the expression of TF targets and identified several key regulators of immune responses. This analysis highlighted both broad shared inflammatory programmes and more selective, context-dependent regulatory responses, with factors such as IRF1 and NFκB marking common activating states and others, including RFXAP and MKX, showing narrower cell type- and stimulus-specific patterns. In particular, IRF1, an established TF downstream of IFN signalling [62], showed strongest inferred activity under IFN-α but was also elevated with IL-2, CytoStim™ and anti-CD3, while inhibitory stimuli (IL-10, IL-4 and TGF-β1) reduced its activity, suggesting that IFN-associated signalling may also be engaged indirectly across multiple stimulation contexts. There was no clear global association between TF expression and downstream TF activity. Whilst some TFs such as IRF1 and STAT3 showed clear positive RNA-activity correlations across cell type-stimulation combinations, the majority of TFs did not. This is likely due to regulatory processes acting beyond mRNA abundance, including post-translational modification, cofactor interactions, dynamic subcellular localisation, and controlled protein turnover [63–67], and demonstrates the value of target-gene-based TF inference. Overall, these results show that stimulation reshapes TF activity across immune cell states through both shared inflammatory axes and more selective cell type-specific regulatory programmes. Further efforts to profile alternate regulators of transcription, such as chromatin states and small non-coding RNAs, at the single-cell level could uncover further layers of control influencing immune responses.

As a demonstration of the translational potential of the stimuli profiles, we performed example analyses to evaluate how well each in vitro stimulus recapitulated data from immune disease samples and in vivo model systems. Comparing our data to in vivo mouse cytokine response data from the "Immune Dictionary" [17] showed that IFN-α responses were highly conserved across both, particularly in myeloid and T cells. However, discrepancies were noted for all other cytokines tested in both, such as IL-2 and TGF-β1. This highlights the complementary value of in vitro and in vivo models and the need for comprehensive profiling of these systems. Divergence between these data sets was not unexpected given differences in their experimental approaches, including cell culture, durations of stimulation and species. Better alignment between in vitro and in vivo data might be expected with mice bearing humanised leukocytes, which can respond similarly to in vitro PBMCs albeit with some differences [68].

Comparing the in vitro stimuli to patient data showed that multiple stimuli reproduce transcriptional changes observed in immune-mediated disease. As expected, IFN-α upregulated SLE-relevant genes including *BST2* across multiple cell types. Conversely, inhibitory conditions like TGF-β1 and IL-10 downregulated SLE disease activation signatures, consistent with their roles as anti-inflammatory mediators. The type I interferon-stimulated gene response in SLE is well documented across many cell types, with both functional changes in monocytes and compositional changes in the CD4^+^ T cell compartment [2, 18, 69]. Identifying key interactions between disease-associated cells in this context could offer new avenues of therapeutic intervention. At the network level, IFN-α showed the greatest overlap with SLE, while LPS and anti-CD3 reproduced more restricted aspects of the disease-associated interaction landscape. However, our analyses also highlighted molecular changes in SLE that were not recapitulated by any in vitro stimulation, such as transcriptional shifts in peripheral MAIT cells. Future work generating similar reference data using patient-derived samples under different stimuli, whilst more limited in scope, may better reproduce such cell types and states that are absent from healthy stimulated samples. Furthermore, comparing these reference data with different patient subpopulations (e.g. stratifying SLE by clinical severity score or comorbidities) may highlight differences in how well particular in vitro models align with distinct patient groups. In general, the use of in vitro stimuli as models of disease necessitates a thorough understanding of both disease and model biology to select appropriate systems; these data should facilitate the latter.

This study is limited to PBMC samples and therefore excludes other inflammatory cell populations present in whole blood, such as granulocytes, platelets and erythroid cells. It is also unlikely to capture tissue-resident disease biology, where cellular composition and spatial context differ substantially from peripheral blood. These are inherent limitations of peripheral blood-based model systems. The present study also captures only a single snapshot of transcriptional dynamics at one time point; longitudinal stimulation profiling could be particularly informative for uncovering regulatory dynamics over time. Exploring additional stimuli, including additional cytokines or pathogen-associated molecular patterns [70, 71] may be necessary to capture the full complexity of disease biology. Combinations of perturbations may also better approximate disease programmes, but their effects are unlikely to be simply additive, and systematic empirical evaluation of all combinations would require high-throughput or automated approaches to perturbation studies [72, 73]. The current dataset may nevertheless prove useful for prioritising candidate combinations based on shared and unique features and overlaps with target diseases of interest.

## Conclusions

The single-cell profiles of PBMC responses to in vitro stimuli presented here provide a framework for understanding the effects of each stimulus on cell type composition, intracellular transcriptional regulation, and intercellular communication. As demonstrated by our analyses, these reference data can be used to efficiently assess the relevance of in vitro models for recapitulating key features of human disease. Together, these features provide a foundational resource for accelerating immunological research and the development of immune-targeted therapies.

## Supplementary Information

Additional file 1.

Additional file 2.

Additional file 3.

Additional file 4.

Additional file 5.

## Data Availability

Raw and processed scRNA-seq data generated during this study have been deposited in the Gene Expression Omnibus under accession GSE327907 (https://www.ncbi.nlm.nih.gov/geo/query/acc.cgi?acc=GSE327907) [74]. A processed scRNA-seq Seurat object is also archived at Zenodo (https://doi.org/10.5281/zenodo.15744698) [75]. Bulk RNA-seq raw and processed data generated during this study have been deposited in the Gene Expression Omnibus under accession GSE327707 (https://www.ncbi.nlm.nih.gov/geo/query/acc.cgi?acc=GSE327707) [76]. Analysis code is available on GitHub (https://github.com/ab-gd1/PBMC_stimulation_manuscript) [77]. Publicly available ImmunexUT bulk RNA-seq data are available from the NBDC Human Database under accession E-GEAD-397 (https://humandbs.dbcls.jp/en/hum0214-v10) [37]. Publicly available SLE scRNA-seq data are available from the Gene Expression Omnibus under accession GSE174188 (https://www.ncbi.nlm.nih.gov/geo/query/acc.cgi?acc=GSE174188) [38]; a processed version obtained from BioTuring [39] was used in this study. Publicly available scRNA-seq data from cytokine-treated mice are available from the Gene Expression Omnibus under accession GSE202186 (https://www.ncbi.nlm.nih.gov/geo/query/acc.cgi?acc=GSE202186) [40].

## Abbreviations

ANCA: Anti-neutrophil cytoplasmic antibody
BCS: B cell stimulator cell line
CD: Cluster of differentiation
cDC: Conventional dendritic cell
DEG: Differentially expressed gene
IFN: Interferon
IL: Interleukin
ILC: Innate lymphoid cell
LPS: Lipopolysaccharide
MAIT: Mucosal-associated invariant T cell
MHC: Major histocompatibility complex
NK: Natural killer cell
PBMC: Peripheral blood mononuclear cell
pDC: Plasmacytoid dendritic cell
RA: Rheumatoid arthritis
RNA: Ribonucleic acid
scRNA-seq: Single-cell RNA sequencing
SLE: Systemic lupus erythematosus
SSc: Systemic sclerosis
TF: Transcription factor
TGF: Transforming growth factor
TNF: Tumour necrosis factor

## References

[1] Kleinstreuer NC, Yang J, Berg EL, Knudsen TB, Richard AM, Martin MT, et al. Phenotypic screening of the ToxCast chemical library to classify toxic and therapeutic mechanisms. Nat Biotechnol. 2014;32(6):583–91. 10.1038/nbt.2914 24837663

[2] Pascual V, Farkas L, Banchereau J. Systemic lupus erythematosus: all roads lead to type I interferons. Curr Opin Immunol. 2006;18(6):676–82. 10.1016/j.coi.2006.09.014 17011763

[3] Duan T, Du Y, Xing C, Wang HY, Wang RF. Toll-like receptor signaling and its role in cell-mediated immunity. Front Immunol. 2022;13:812774. 10.3389/fimmu.2022.812774 PMC8927970 35309296

[4] Jang DI, Lee AH, Shin HY, Song HR, Park JH, Kang TB, et al. The role of Tumor Necrosis Factor Alpha (TNF-alpha) in autoimmune disease and current TNF-alpha Inhibitors in therapeutics. Int J Mol Sci. 2021;22(5):2719 . 10.3390/ijms22052719 PMC7962638 33800290

[5] Reyes M, Leff SM, Gentili M, Hacohen N, Blainey PC. Microscale combinatorial stimulation of human myeloid cells reveals inflammatory priming by viral ligands. Sci Adv. 2023;9(8):eade5090. 10.1126/sciadv.ade5090 PMC9956118 36827376

[6] Montaldo E, Lusito E, Bianchessi V, Caronni N, Scala S, Basso-Ricci L, et al. Cellular and transcriptional dynamics of human neutrophils at steady state and upon stress. Nat Immunol. 2022;23(10):1470–83. 10.1038/s41590-022-01311-1 PMC7615267 36138183

[7] Wendisch D, Dietrich O, Mari T, von Stillfried S, Ibarra IL, Mittermaier M, et al. SARS-CoV-2 infection triggers profibrotic macrophage responses and lung fibrosis. Cell. 2021;184(26):6243-61 e27. 10.1016/j.cell.2021.11.033 PMC8626230 34914922

[8] Fairfax BP, Humburg P, Makino S, Naranbhai V, Wong D, Lau E, et al. Innate immune activity conditions the effect of regulatory variants upon monocyte gene expression. Science. 2014;343(6175):1246949. 10.1126/science.1246949 PMC4064786 24604202

[9] van der Heijden WA, Van de Wijer L, Keramati F, Trypsteen W, Rutsaert S, Horst RT, et al. Chronic HIV infection induces transcriptional and functional reprogramming of innate immune cells. JCI Insight. 2021. 10.1172/jci.insight.145928. PMC8119206 33630761

[10] Cano-Gamez E, Soskic B, Roumeliotis TI, So E, Smyth DJ, Baldrighi M, et al. Single-cell transcriptomics identifies an effectorness gradient shaping the response of CD4(+) T cells to cytokines. Nat Commun. 2020;11(1):1801. 10.1038/s41467-020-15543-y PMC7156481 32286271

[11] Szabo PA, Levitin HM, Miron M, Snyder ME, Senda T, Yuan J, et al. Single-cell transcriptomics of human T cells reveals tissue and activation signatures in health and disease. Nat Commun. 2019;10(1):4706. 10.1038/s41467-019-12464-3 PMC6797728 31624246

[12] Derbois C, Palomares MA, Deleuze JF, Cabannes E, Bonnet E. Single cell transcriptome sequencing of stimulated and frozen human peripheral blood mononuclear cells. Sci Data. 2023;10(1):433. 10.1038/s41597-023-02348-z PMC10326076 37414801

[13] Mimitou EP, Lareau CA, Chen KY, Zorzetto-Fernandes AL, Hao Y, Takeshima Y, et al. Scalable, multimodal profiling of chromatin accessibility, gene expression and protein levels in single cells. Nat Biotechnol. 2021;39(10):1246–58. 10.1038/s41587-021-00927-2 PMC8763625 34083792

[14] Lawlor N, Nehar-Belaid D, Grassmann JDS, Stoeckius M, Smibert P, Stitzel ML, et al. Single cell analysis of blood mononuclear cells stimulated through either LPS or Anti-CD3 and Anti-CD28. Front Immunol. 2021;12:636720. 10.3389/fimmu.2021.636720 PMC8010670 33815388

[15] Kartha VK, Duarte FM, Hu Y, Ma S, Chew JG, Lareau CA, et al. Functional inference of gene regulation using single-cell multi-omics. Cell Genom. 2022;2(9):100166. 10.1016/j.xgen.2022.100166 PMC9534481 36204155

[16] Oelen R, de Vries DH, Brugge H, Gordon MG, Vochteloo M, single-cell e Qc, et al. Single-cell RNA-sequencing of peripheral blood mononuclear cells reveals widespread, context-specific gene expression regulation upon pathogenic exposure. Nat Commun. 2022;13(1):3267. 10.1038/s41467-022-30893-5 PMC9174272 35672358

[17] Cui A, Huang T, Li S, Ma A, Pérez JL, Sander C, et al. Dictionary of immune responses to cytokines at single-cell resolution. Nature. 2024;625(7994):377–84. 10.1038/s41586-023-06816-9 PMC10781646 38057668

[18] Perez RK, Gordon MG, Subramaniam M, Kim MC, Hartoularos GC, Targ S, et al. Single-cell RNA-seq reveals cell type-specific molecular and genetic associations to lupus. Science. 2022;376(6589):eabf1970. 10.1126/science.abf1970 PMC9297655 35389781

[19] Ota M, Nagafuchi Y, Hatano H, Ishigaki K, Terao C, Takeshima Y, et al. Dynamic landscape of immune cell-specific gene regulation in immune-mediated diseases. Cell. 2021;184(11):3006-21.e17. 10.1016/j.cell.2021.03.056 33930287

[20] Kopylova E, Noe L, Touzet H. SortMeRNA: fast and accurate filtering of ribosomal RNAs in metatranscriptomic data. Bioinformatics. 2012;28(24):3211–7. 10.1093/bioinformatics/bts611 23071270

[21] Ewels PA, Peltzer A, Fillinger S, Patel H, Alneberg J, Wilm A, et al. The nf-core framework for community-curated bioinformatics pipelines. Nat Biotechnol. 2020;38(3):276–8. 10.1038/s41587-020-0439-x 32055031

[22] Patro R, Duggal G, Love MI, Irizarry RA, Kingsford C. Salmon provides fast and bias-aware quantification of transcript expression. Nat Methods. 2017;14(4):417–9. 10.1038/nmeth.4197 PMC5600148 28263959

[23] Soneson C, Love MI, Robinson MD. Differential analyses for RNA-seq: transcript-level estimates improve gene-level inferences. Res. 2015;4:F1000Res. 10.12688/f1000research.7563.1 PMC4712774 26925227

[24] Love MI, Huber W, Anders S. Moderated estimation of fold change and dispersion for RNA-seq data with DESeq2. Genome Biol. 2014;15(12):550. 10.1186/s13059-014-0550-8 PMC4302049 25516281

[25] Hao Y, Stuart T, Kowalski MH, Choudhary S, Hoffman P, Hartman A, et al. Dictionary learning for integrative, multimodal and scalable single-cell analysis. Nat Biotechnol. 2024;42(2):293–304. 10.1038/s41587-023-01767-y PMC10928517 37231261

[26] Aran D, Looney AP, Liu L, Wu E, Fong V, Hsu A, et al. Reference-based analysis of lung single-cell sequencing reveals a transitional profibrotic macrophage. Nat Immunol. 2019;20(2):163–72. 10.1038/s41590-018-0276-y PMC6340744 30643263

[27] Monaco G, Lee B, Xu W, Mustafah S, Hwang YY, Carré C, et al. RNA-Seq signatures normalized by mRNA abundance allow absolute deconvolution of human immune cell types. Cell Rep. 2019;26(6):1627-40.e7. 10.1016/j.celrep.2019.01.041 PMC6367568 30726743

[28] McGinnis CS, Murrow LM, Gartner ZJ. DoubletFinder: doublet detection in single-cell RNA sequencing data using artificial nearest neighbors. Cell Syst. 2019;8(4):329-37.e4. 10.1016/j.cels.2019.03.003 PMC6853612 30954475

[29] Hao Y, Hao S, Andersen-Nissen E, Mauck WM, Zheng S, Butler A, et al. Integrated analysis of multimodal single-cell data. Cell. 2021;184(13):3573-87.e29. 10.1016/j.cell.2021.04.048 PMC8238499 34062119

[30] Phipson B, Sim CB, Porrello ER, Hewitt AW, Powell J, Oshlack A. Propeller: testing for differences in cell type proportions in single cell data. Bioinformatics. 2022;38(20):4720–6. 10.1093/bioinformatics/btac582 PMC9563678 36005887

[31] Korotkevich G, Sukhov V, Budin N, Shpak B, Artyomov MN, Sergushichev A. Fast gene set enrichment analysis. bioRxiv. 2021:060012.

[32] Milacic M, Beavers D, Conley P, Gong C, Gillespie M, Griss J, et al. The Reactome pathway knowledgebase 2024. Nucleic Acids Res. 2024;52(D1):D672–8. 10.1093/nar/gkad1025 PMC10767911 37941124

[33] Browaeys R, Gilis J, Sang-Aram C, De Bleser P, Hoste L, Tavernier S, et al. MultiNicheNet: a flexible framework for differential cell-cell communication analysis from multi-sample multi-condition single-cell transcriptomics data. bioRxiv. 2023:2023.06.13.544751.

[34] Badia IMP, Velez Santiago J, Braunger J, Geiss C, Dimitrov D, Muller-Dott S, et al. decoupleR: ensemble of computational methods to infer biological activities from omics data. Bioinform Adv. 2022;2(1):vbac016. 10.1093/bioadv/vbac016 PMC9710656 36699385

[35] Muller-Dott S, Tsirvouli E, Vazquez M, Ramirez Flores RO, Badia IMP, Fallegger R, et al. Expanding the coverage of regulons from high-confidence prior knowledge for accurate estimation of transcription factor activities. Nucleic Acids Res. 2023;51(20):10934–49. 10.1093/nar/gkad841 PMC10639077 37843125

[36] Ritchie ME, Phipson B, Wu D, Hu Y, Law CW, Shi W, et al. Limma powers differential expression analyses for RNA-sequencing and microarray studies. Nucleic Acids Res. 2015;43(7):e47. 10.1093/nar/gkv007 PMC4402510 25605792

[37] Ota M, Nagafuchi Y, Hatano H, Ishigaki K, Terao C, Takeshima Y, et al. ImmuNexUT transcriptome dataset. Datasets. NBDC Human Database. 2021. https://humandbs.dbcls.jp/en/hum0214-v10.

[38] Perez RK, Gordon MG, Subramaniam M, Kim MC, Hartoularos G, Targ S, et al. Multiplexed scRNA-seq reveals the cellular and genetic correlates of systemic lupus erythematosus. Datasets. Gene Expression Omnibus. 2021. https://www.ncbi.nlm.nih.gov/geo/query/acc.cgi?acc=GSE174188.

[39] BioTuring. B browserX. https://bioturing.com/single-cell-analysis-bbrowserx

[40] Cui A, Huang T, Li S, Ma A, Perez JL, Sander C, et al. Single-cell RNA-seq dictionary of in vivo immune responses to cytokines. Datasets Gene Expression Omnibus. 2023. https://www.ncbi.nlm.nih.gov/geo/query/acc.cgi?acc=GSE202186.

[41] NCBI. HomoloGene. http://www.ncbi.nlm.nih.gov/homologene

[42] Durinck S, Spellman PT, Birney E, Huber W. Mapping identifiers for the integration of genomic datasets with the R/Bioconductor package biomaRt. Nat Protoc. 2009;4(8):1184–91. 10.1038/nprot.2009.97 PMC3159387 19617889

[43] Wolk K, Döcke W-D, von Baehr V, Volk H-D, Sabat R. Impaired antigen presentation by human monocytes during endotoxin tolerance. Blood. 2000;96(1):218–23. 10891454

[44] Fisher J, Wood O, Bullers S, Murray L, Li L, Jackson-Wood MA. Machine learning predictions surpass individual mRNAs as a proxy of single-cell protein expression. Genome Biol. 2026. 10.1186/s13059-026-04083-1. PMC13235196 42021407

[45] Korsmeyer SJ. BCL-2 gene family and the regulation of programmed cell death. Cancer Res. 1999;59(7 Suppl):1693s-s1700. 10197582

[46] Cobaleda C, Schebesta A, Delogu A, Busslinger M. Pax5: the guardian of B cell identity and function. Nat Immunol. 2007;8(5):463–70. 10.1038/ni1454 17440452

[47] Sugimoto N, Oida T, Hirota K, Nakamura K, Nomura T, Uchiyama T, et al. Foxp3-dependent and -independent molecules specific for CD25+CD4+ natural regulatory T cells revealed by DNA microarray analysis. Int Immunol. 2006;18(8):1197–209. 10.1093/intimm/dxl060 16772372

[48] Li Y, Qi X, Liu B, Huang H. The STAT5-GATA2 pathway is critical in basophil and mast cell differentiation and maintenance. J Immunol. 2015;194(9):4328–38. 10.4049/jimmunol.1500018 PMC4405376 25801432

[49] Wu X, Satpathy AT, Kc W, Liu P, Murphy TL, Murphy KM. Bcl11a controls Flt3 expression in early hematopoietic progenitors and is required for pDC development in vivo. PLoS ONE. 2013;8(5):e64800. 10.1371/journal.pone.0064800 PMC3669380 23741395

[50] Durand B, Sperisen P, Emery P, Barras E, Zufferey M, Mach B, et al. RFXAP, a novel subunit of the RFX DNA binding complex is mutated in MHC class II deficiency. EMBO J. 1997;16(5):1045–55. 10.1093/emboj/16.5.1045 PMC1169704 9118943

[51] Lynch CA, Acosta SA, Anderson DM, Rogers GE, Wilson-Rawls J, Rawls A. The transcription factor Mohawk facilitates skeletal muscle repair via modulation of the inflammatory environment. Int J Mol Sci. 2024. 10.3390/ijms25095019. PMC11084535 38732238

[52] Tecalco-Cruz AC, Sosa-Garrocho M, Vazquez-Victorio G, Ortiz-Garcia L, Dominguez-Huttinger E, Macias-Silva M. Transforming growth factor-beta/SMAD Target gene SKIL is negatively regulated by the transcriptional cofactor complex SNON-SMAD4. J Biol Chem. 2012;287(32):26764–76. 10.1074/jbc.M112.386599 PMC3411014 22674574

[53] Bissey PA, Law JH, Bruce JP, Shi W, Renoult A, Chua MLK, et al. Dysregulation of the MiR-449b target TGFBI alters the TGFbeta pathway to induce cisplatin resistance in nasopharyngeal carcinoma. Oncogenesis. 2018;7(5):40. 10.1038/s41389-018-0050-x PMC5966388 29795279

[54] Chiba A, Tamura N, Yoshikiyo K, Murayama G, Kitagaichi M, Yamaji K, et al. Activation status of mucosal-associated invariant T cells reflects disease activity and pathology of systemic lupus erythematosus. Arthritis Res Ther. 2017;19(1):58. 10.1186/s13075-017-1257-5 PMC5348792 28288675

[55] Bankole S, Ng LG, Ginhoux F. Multi-talented myeloid cells. Discov Immunol. 2025;4(1):kyaf015. 10.1093/discim/kyaf015 PMC12770966 41503142

[56] Batten M, Kljavin NM, Li J, Walter MJ, de Sauvage FJ, Ghilardi N. Cutting edge: IL-27 is a potent inducer of IL-10 but not FoxP3 in murine T cells. J Immunol. 2008;180(5):2752–6. 10.4049/jimmunol.180.5.2752 18292493

[57] Andrews C, McLean MH, Durum SK. Interleukin-27 as a novel therapy for inflammatory bowel disease: a critical review of the literature. Inflamm Bowel Dis. 2016;22(9):2255–64. 10.1097/MIB.0000000000000818 PMC4992429 27243591

[58] Lastres P, Letamendia A, Zhang H, Rius C, Almendro N, Raab U, et al. Endoglin modulates cellular responses to TGF-beta 1. J Cell Biol. 1996;133(5):1109–21. 10.1083/jcb.133.5.1109 PMC2120857 8655583

[59] Tomayko MM, Karaaslan S, Lainez B, Conter LJ, Song E, Venkatesan S, et al. Roles of bone morphogenetic protein receptor 1A in germinal centers and long-lived humoral immunity. Immunohorizons. 2021;5(5):284–97. 10.4049/immunohorizons.2100019 PMC12221147 33975878

[60] Akdis M, Aab A, Altunbulakli C, Azkur K, Costa RA, Crameri R, et al. Interleukins (from IL-1 to IL-38), interferons, transforming growth factor β, and TNF-α: receptors, functions, and roles in diseases. J Allergy Clin Immunol. 2016;138(4):984–1010. 10.1016/j.jaci.2016.06.033 27577879

[61] Chen KY, Takeshima Y, Zorzetto-Fernandes AL, Makino K, Kibayashi T, Hata M, et al. Joint single-cell measurements of surface proteins, intracellular proteins and gene expression with icCITE-seq. bioRxiv. 2025:2025.01.11.632564.

[62] Man SM, Karki R, Malireddi RK, Neale G, Vogel P, Yamamoto M, et al. The transcription factor IRF1 and guanylate-binding proteins target activation of the AIM2 inflammasome by *Francisella* infection. Nat Immunol. 2015;16(5):467–75. 10.1038/ni.3118 PMC4406811 25774715

[63] Larsen FB, Kragelund BB, Lindorff-Larsen K, Heidarsson PO, Hartmann-Petersen R. Proteasomal control of transcription factors: mechanisms, regulation and dysregulation. Cell Mol Life Sci. 2026;83(1):142. 10.1007/s00018-026-06103-6 PMC12979741 41764121

[64] Veitia RA. Rethinking transcription factor dynamics and transcription regulation in eukaryotes. Trends Biochem Sci. 2025;50(5):376–84. 10.1016/j.tibs.2025.01.012 40044550

[65] Pan D. The hippo signaling pathway in development and cancer. Dev Cell. 2010;19(4):491–505. 10.1016/j.devcel.2010.09.011 PMC3124840 20951342

[66] Fan CM, Maniatis T. Generation of p50 subunit of NF-kappa B by processing of p105 through an ATP-dependent pathway. Nature. 1991;354(6352):395–8. 10.1038/354395a0 1956402

[67] Farrell AS, Sears RC. MYC degradation. Cold Spring Harb Perspect Med. 2014;4(3):a014365. 10.1101/cshperspect.a014365 PMC3935390 24591536

[68] Cheng L, Zhang Z, Li G, Li F, Wang L, Zhang L, et al. Human innate responses and adjuvant activity of TLR ligands in vivo in mice reconstituted with a human immune system. Vaccine. 2017;35(45):6143–53. 10.1016/j.vaccine.2017.09.052 PMC5641266 28958808

[69] Nehar-Belaid D, Hong S, Marches R, Chen G, Bolisetty M, Baisch J, et al. Mapping systemic lupus erythematosus heterogeneity at the single-cell level. Nat Immunol. 2020;21(9):1094–106. 10.1038/s41590-020-0743-0 PMC7442743 32747814

[70] Serrano R, Wesch D, Kabelitz D. Correction: Serrano, R.; Wesch, D, Kabelitz, D. Activation of human gammadelta T cells: modulation by toll-like receptor 8 ligands and role of monocytes. Cells 2020;9(9):713. 10.3390/cells9030713 PMC7140608 32183240

[71] Vlaming KE, van Wijnbergen K, Kaptein TM, Nijhuis M, Kootstra NJ, de Bree GJ, et al. Crosstalk between TLR8 and RIG-I-like receptors enhances antiviral immune responses. Front Med (Lausanne). 2023;10:1146457. 10.3389/fmed.2023.1146457 PMC10227620 37261119

[72] Fleige L, Capellino S. Simultaneous stimulation of peripheral blood mononuclear cells with CpG ODN2006 and alpha-IgM antibodies leads to strong immune responses in monocytes independent of B Cell Activation. Cells. 2024;13(22):1822. 10.3390/cells13221822 PMC11593001 39594572

[73] Glass MC, Glass DR, Oliveria JP, Mbiribindi B, Esquivel CO, Krams SM, et al. Human IL-10-producing B cells have diverse states that are induced from multiple B cell subsets. Cell Rep. 2022;39(3):110728. 10.1016/j.celrep.2022.110728 PMC9107325 35443184

[74] Wood O, Braithwaite AT, Fisher J, Li L, Murray L, Paluch C, et al. Integrated single‑cell transcriptomic profiling of human peripheral blood mononuclear cells across diverse in vitro immune stimuli. Datasets. Gene Expression Omnibus. 2025. https://www.ncbi.nlm.nih.gov/geo/query/acc.cgi?acc=GSE327907.

[75] Wood O, Braithwaite AT, Fisher J, Li L, Murray L, Paluch C, et al. Intra and intercellular immune responses across diverse in vitro stimuli and inflammatory disease. Datasets. Zenodo. 2025. 10.5281/zenodo.15744698 PMC13651199 42458559

[76] Wood O, Braithwaite AT, Fisher J, Li L, Murray L, Paluch C, et al. Sorted bulk RNA-seq profiling of human peripheral blood mononuclear cell populations across diverse in vitro immune stimuli. Datasets. Gene Expression Omnibus. 2025. https://www.ncbi.nlm.nih.gov/geo/query/acc.cgi?acc=GSE327707

[77] Braithwaite AT. Code for: A map of intra- and intercellular immune responses across diverse in vitro stimuli and inflammatory disease. GitHub. 2025. https://github.com/ab-gd1/PBMC_stimulation_manuscript. 10.1186/s13073-026-01721-y PMC13651199 42458559

