# Supplementary material for: A map of intra- and intercellular immune responses across diverse in vitro stimuli and inflammatory disease
Source: Genome Med. 2026 Jul 15;18:148. doi: 10.1186/s13073-026-01721-y (PMC13651199; doi:10.1186/s13073-026-01721-y)
Supplement: Supplementary file 1 [file 13073_2026_1721_MOESM1_ESM.pdf]

# Figure S1

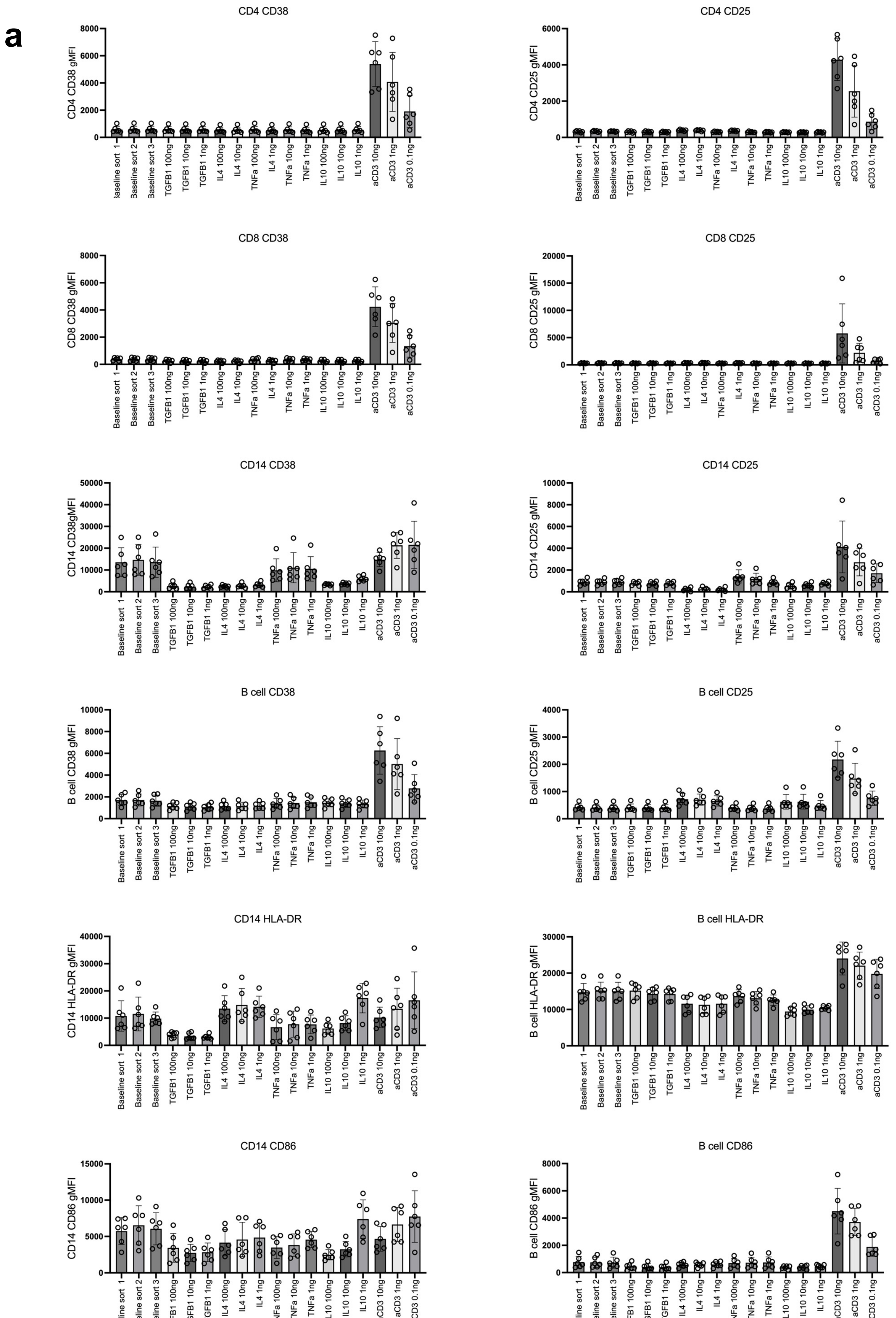
a
CD4 CD38
CD4 CD25
CD8 CD38
CD8 CD25
CD14 CD38
CD14 CD25
B cell CD38
B cell CD25
CD14 HLA-DR
B cell HLA-DR
CD14 CD86
B cell CD86
Baseline sort 1
Baseline sort 2
Baseline sort 3
TGFB1 100ng
TGFB1 10ng
TGFB1 1ng
IL4 100ng
IL4 10ng
IL4 1ng
TNFa 100ng
TNFa 10ng
TNFa 1ng
IL10 100ng
IL10 10ng
IL10 1ng
aCD3 10ng
aCD3 1ng
aCD3 0.1ng

**Figure S1**

**b**

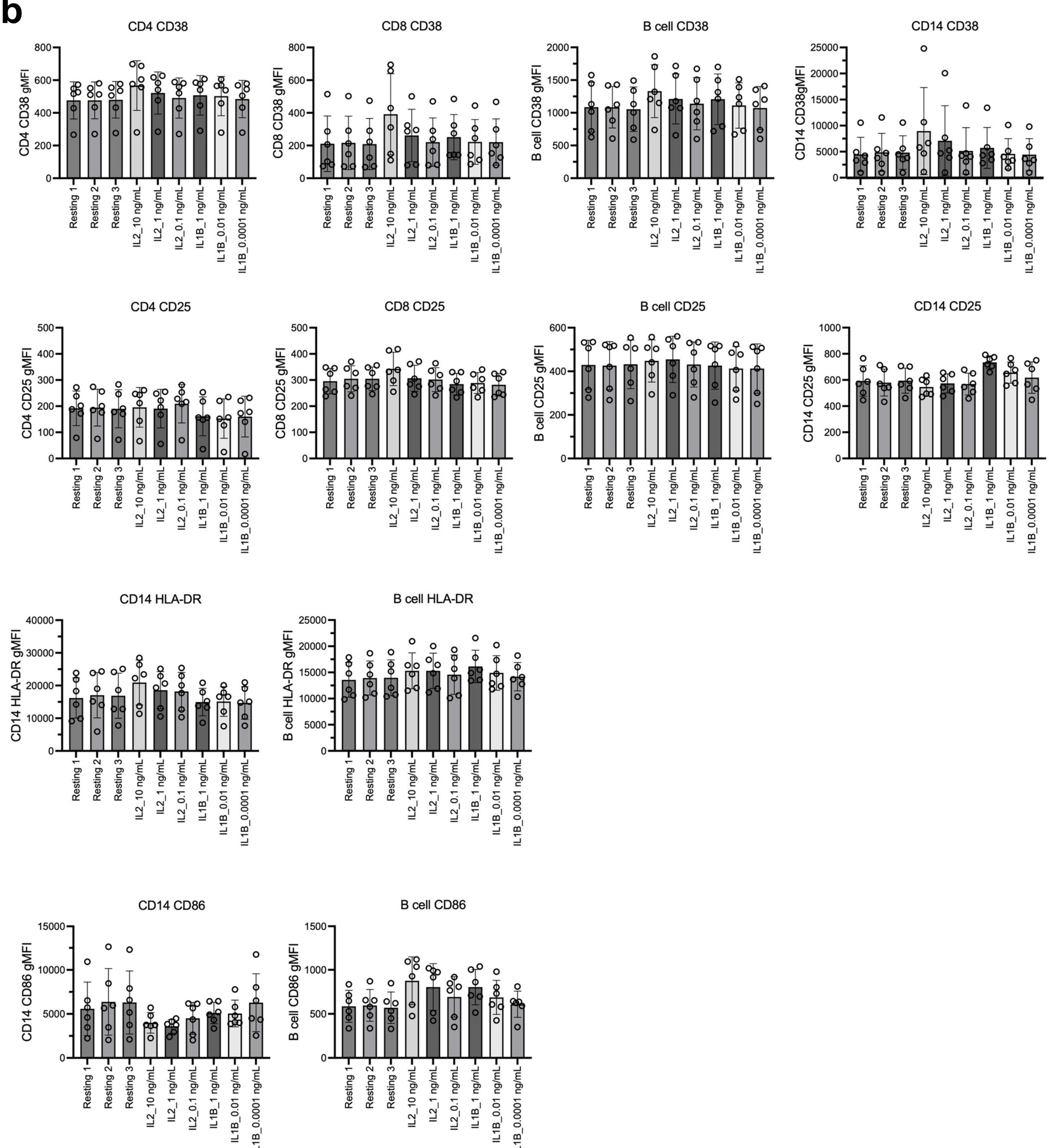
CD4 CD38
CD4 CD38 gMFI
CD8 CD38
CD8 CD38 gMFI
B cell CD38
B cell CD38 gMFI
CD14 CD38
CD14 CD38gMFI
CD4 CD25
CD4 CD25 gMFI
CD8 CD25
CD8 CD25 gMFI
B cell CD25
B cell CD25 gMFI
CD14 CD25
CD14 CD25 gMFI
CD14 HLA-DR
CD14 HLA-DR gMFI
B cell HLA-DR
B cell HLA-DR gMFI
CD14 CD86
CD14 CD86 gMFI
B cell CD86
B cell CD86 gMFI
Resting 1
Resting 2
Resting 3
IL2_10 ng/mL
IL2_1 ng/mL
IL2_0.1 ng/mL
IL1B_1 ng/mL
IL1B_0.01 ng/mL
IL1B_0.0001 ng/mL

**Figure S1**

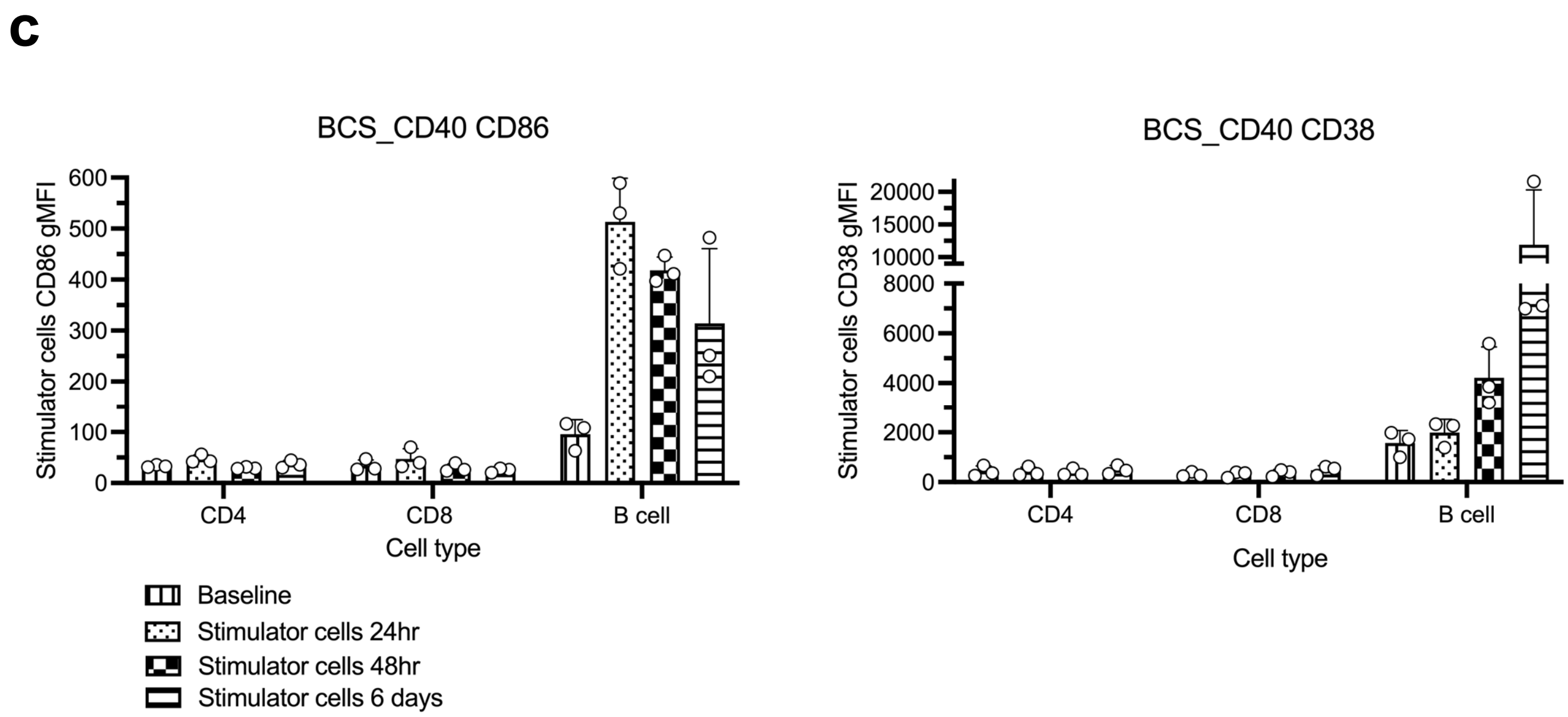
c
BCS_CD40 CD86
Stimulator cells CD86 gMFI
600
500
400
300
200
100
0
CD4
CD8
B cell
Cell type
BCS_CD40 CD38
Stimulator cells CD38 gMFI
20000
15000
10000
8000
6000
4000
2000
0
CD4
CD8
B cell
Cell type
Baseline
Stimulator cells 24hr
Stimulator cells 48hr
Stimulator cells 6 days

**Figure S1**

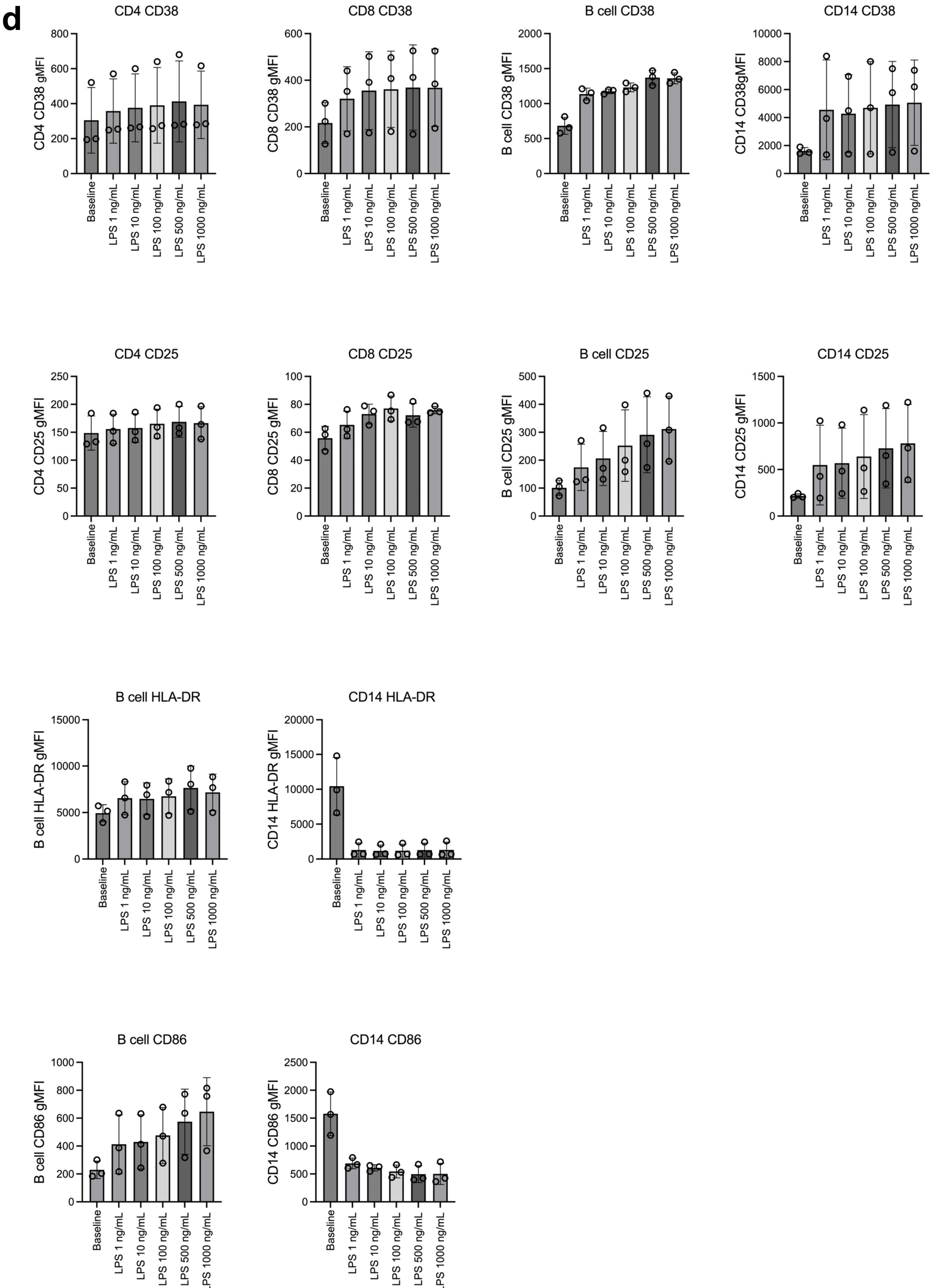
d
CD4 CD38
CD4 CD38 gMFI
CD8 CD38
CD8 CD38 gMFI
B cell CD38
B cell CD38 gMFI
CD14 CD38
CD14 CD38gMFI
CD4 CD25
CD4 CD25 gMFI
CD8 CD25
CD8 CD25 gMFI
B cell CD25
B cell CD25 gMFI
CD14 CD25
CD14 CD25 gMFI
B cell HLA-DR
B cell HLA-DR gMFI
CD14 HLA-DR
CD14 HLA-DR gMFI
B cell CD86
B cell CD86 gMFI
CD14 CD86
CD14 CD86 gMFI
Baseline
LPS 1 ng/mL
LPS 10 ng/mL
LPS 100 ng/mL
LPS 500 ng/mL
LPS 1000 ng/mL

**Figure S1**
Validation experiments to determine the concentration of immunomodulators used for the *in vitro* stimulation. A range of concentrations were tested, the final selection was based on evidence of an effect and / or retention of cell type frequencies after 48 hours of in vitro culture. (A) Titration of stimulation conditions TGFB1, IL4, TNFa, IL10, anti-CD3. (B) Titration of stimulation conditions IL2 and IL1B. (C) B cell stimulator (BCS) time course. (D) Titration of LPS condition.

**Figure S2**

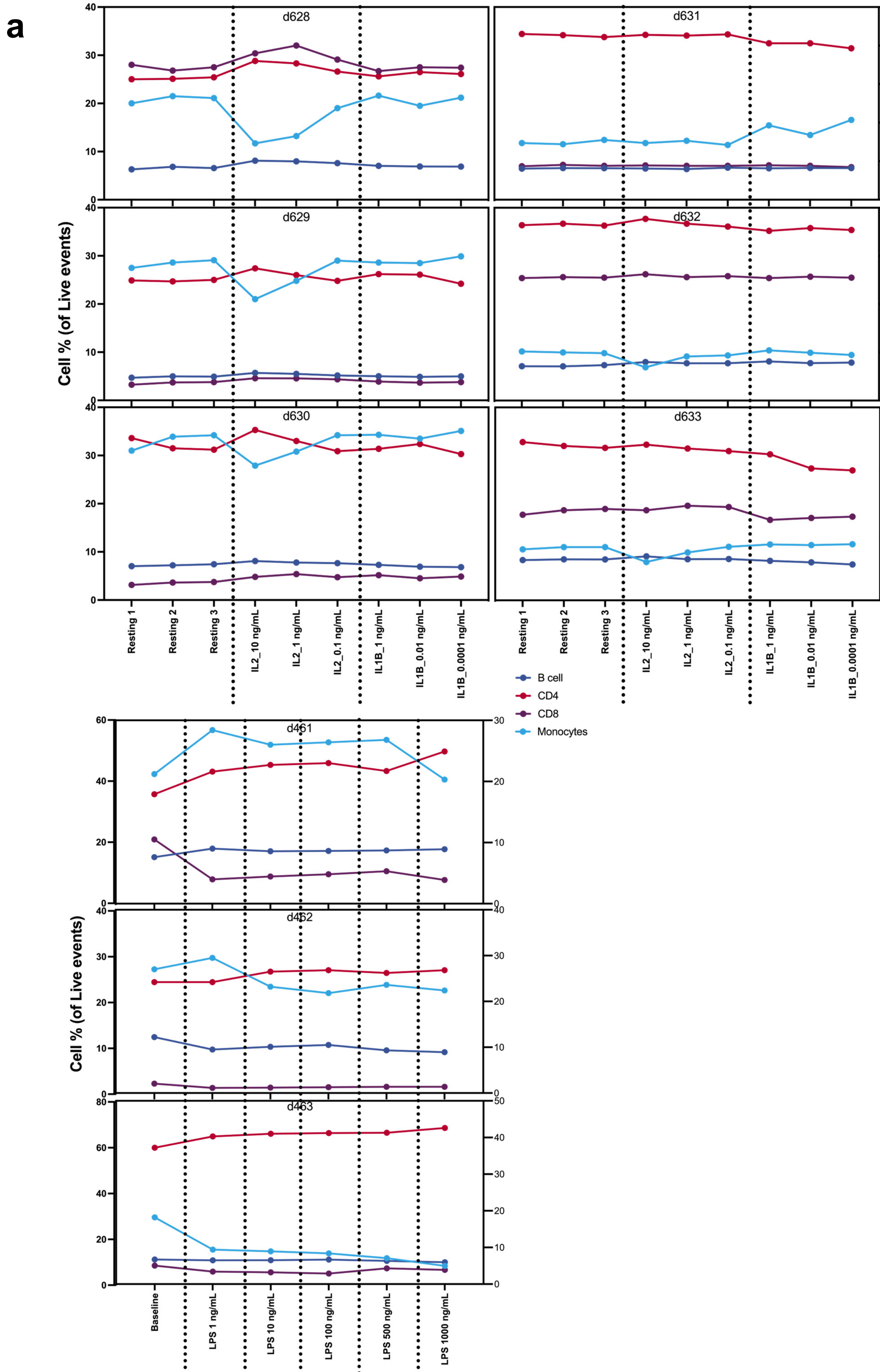

a
d628
d629
d630
d631
d632
d633
d461
d462
d463
Cell % (of Live events)
Resting 1
Resting 2
Resting 3
IL2_10 ng/mL
IL2_1 ng/mL
IL2_0.1 ng/mL
IL1B_1 ng/mL
IL1B_0.01 ng/mL
IL1B_0.0001 ng/mL
Baseline
LPS 1 ng/mL
LPS 10 ng/mL
LPS 100 ng/mL
LPS 500 ng/mL
LPS 1000 ng/mL
B cell
CD4
CD8
Monocytes

Figure S2

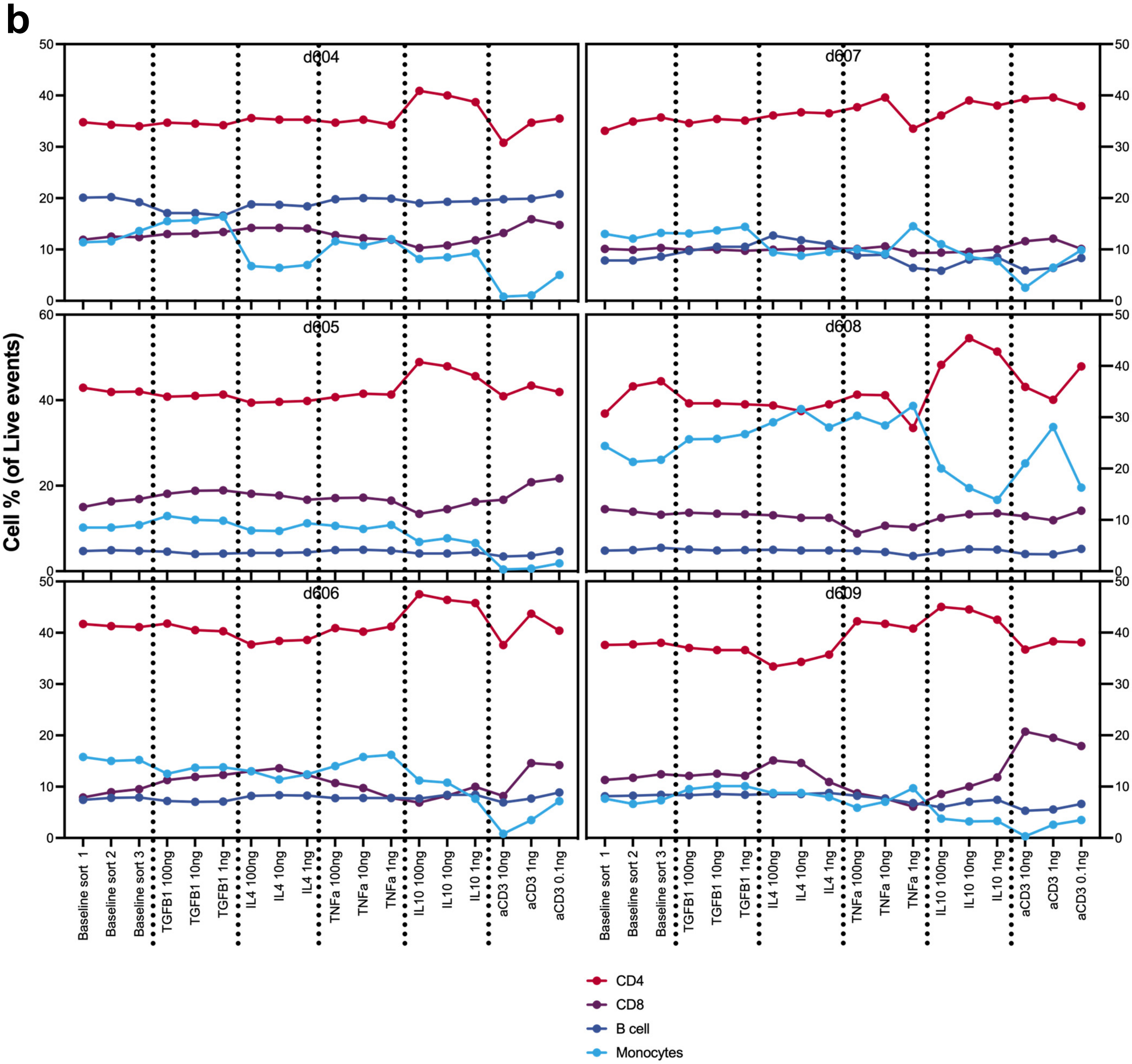
b
d604
d607
d605
d608
d606
d609
Cell % (of Live events)
Baseline sort 1
Baseline sort 2
Baseline sort 3
TGFB1 100ng
TGFB1 10ng
TGFB1 1ng
IL4 100ng
IL4 10ng
IL4 1ng
TNFa 100ng
TNFa 10ng
TNFa 1ng
IL10 100ng
IL10 10ng
IL10 1ng
aCD3 10ng
aCD3 1ng
aCD3 0.1ng
CD4
CD8
B cell
Monocytes

**Figure S2**

**c**

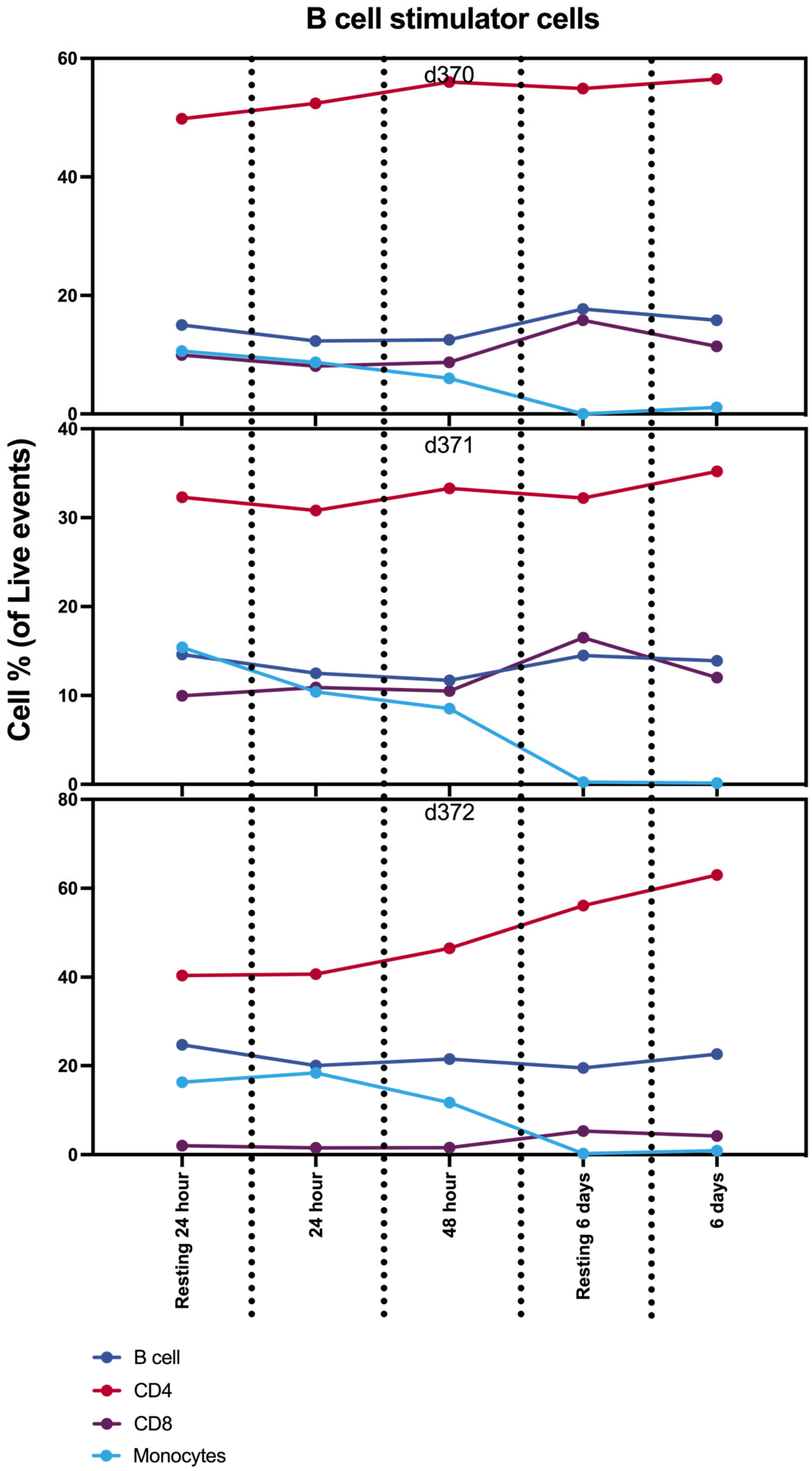

B cell stimulator cells
d370
d371
d372
Cell % (of Live events)
60
40
20
0
40
30
20
10
0
80
60
40
20
0
Resting 24 hour
24 hour
48 hour
Resting 6 days
6 days
B cell
CD4
CD8
Monocytes

**Figure S2**
Changes in cellular composition associated with the titration and timecourse of immunomodulators. The % of live events for CD4 and CD8 T cells, B cell and Myeloid cells are plotted across donors. (A) Titration of stimulation conditions IL2, IL1B and LPS. (B) Titration of stimulation conditions TGFB1, IL4, TNFa, IL10, anti-CD3. (C) B cell stimulator (BCS) time course.

**Figure S3**

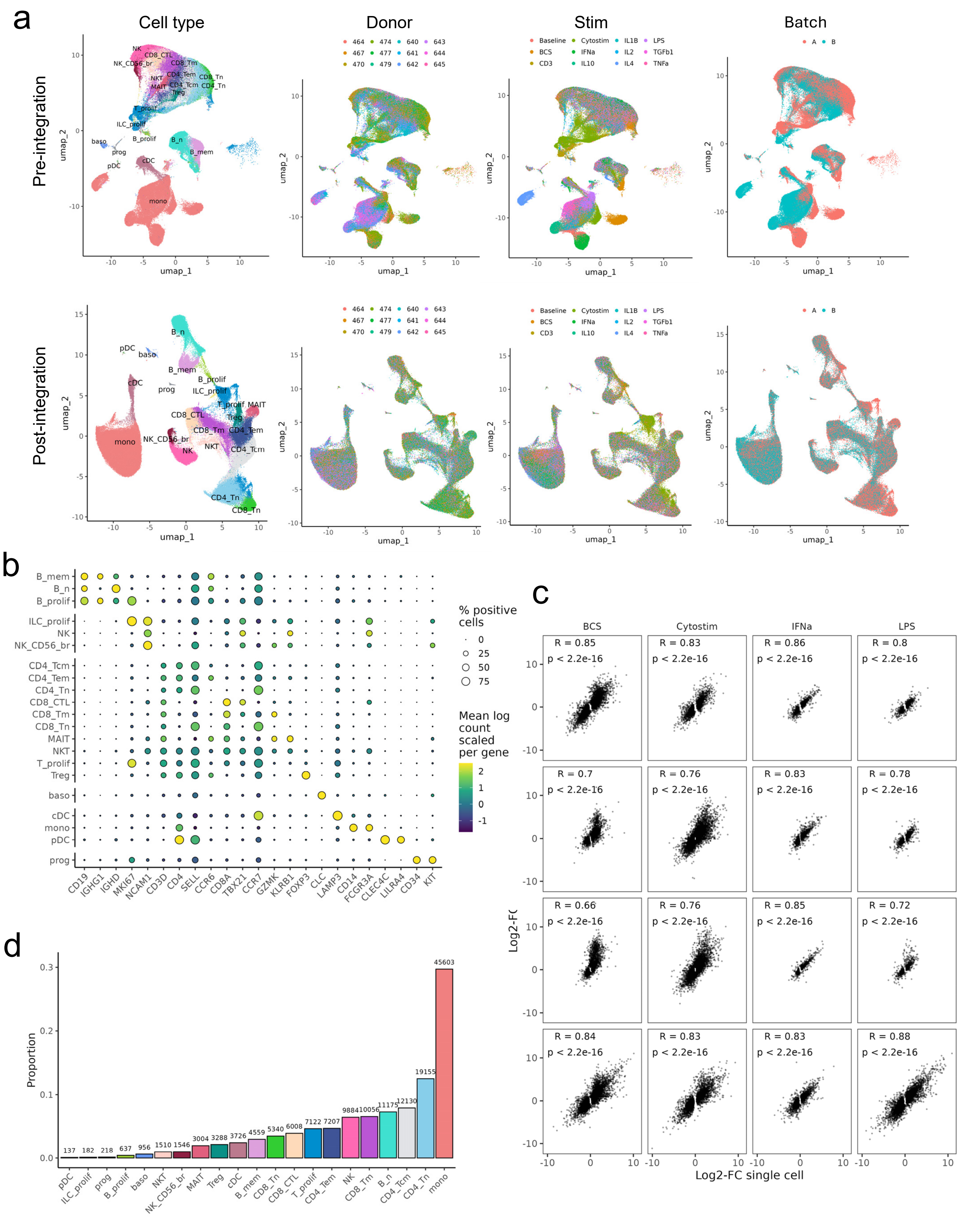

a
Cell type
Donor
Stim
Batch
Pre-integration
Post-integration
umap_1
umap_2
b
% positive cells
Mean log count scaled per gene
c
BCS
Cytostim
IFNa
LPS
Log2-FC
Log2-FC single cell
d
Proportion

# Figure S3

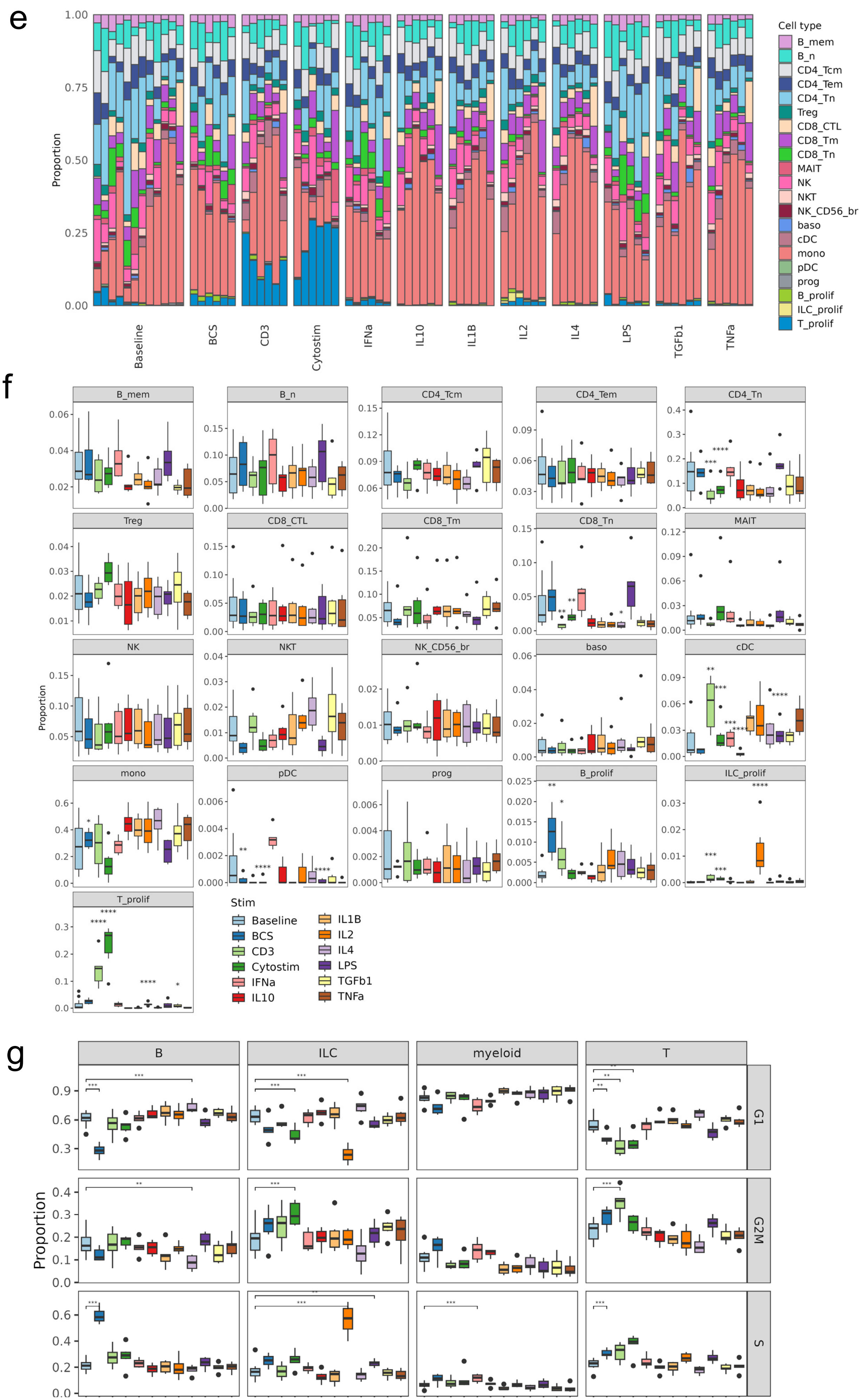

e
Proportion
1.00
0.75
0.50
0.25
0.00
Baseline
BCS
CD3
Cytostim
IFNa
IL10
IL1B
IL2
IL4
LPS
TGFb1
TNFa
Cell type
B_mem
B_n
CD4_Tcm
CD4_Tem
CD4_Tn
Treg
CD8_CTL
CD8_Tm
CD8_Tn
MAIT
NK
NKT
NK_CD56_br
baso
cDC
mono
pDC
prog
B_prolif
ILC_prolif
T_prolif
f
Proportion
B_mem
B_n
CD4_Tcm
CD4_Tem
CD4_Tn
Treg
CD8_CTL
CD8_Tm
CD8_Tn
MAIT
NK
NKT
NK_CD56_br
baso
cDC
mono
pDC
prog
B_prolif
ILC_prolif
T_prolif
Stim
Baseline
BCS
CD3
Cytostim
IFNa
IL10
IL1B
IL2
IL4
LPS
TGFb1
TNFa
g
Proportion
B
ILC
myeloid
T
G1
G2M
S

**Figure S3**

(A) UMAP dimensional reductions of cells by cell type, donor, stimulation condition and batch (pre- and post-integration). (B) Expression of cell type marker genes per final annotation (scaled per gene). (C) Comparison of log2-fold changes (stimulated vs. unstimulated) from sorted bulk populations (bulk RNA-seq) with equivalent subsets from the single cell RNA-seq data. R,p – Pearson correlation of log2-fold changes for all genes with adjusted $p<0.05$ in either dataset. (D) Total number of cells captured for each cell type. (E) Proportion of cell types per donor across each stimulation. (F) Proportion of cell types per donor summarised as box plots across each stimulation with Propeller compositional assessment for each condition vs. baseline. (G) Donor-level summary of cell cycle phase per cell type/stimulation condition with Wilcoxon test for each condition vs. baseline. * adjusted $p<0.05$; ** $p<0.01$; *** $p<0.001$; **** $p<0.001$.

**Figure S4**

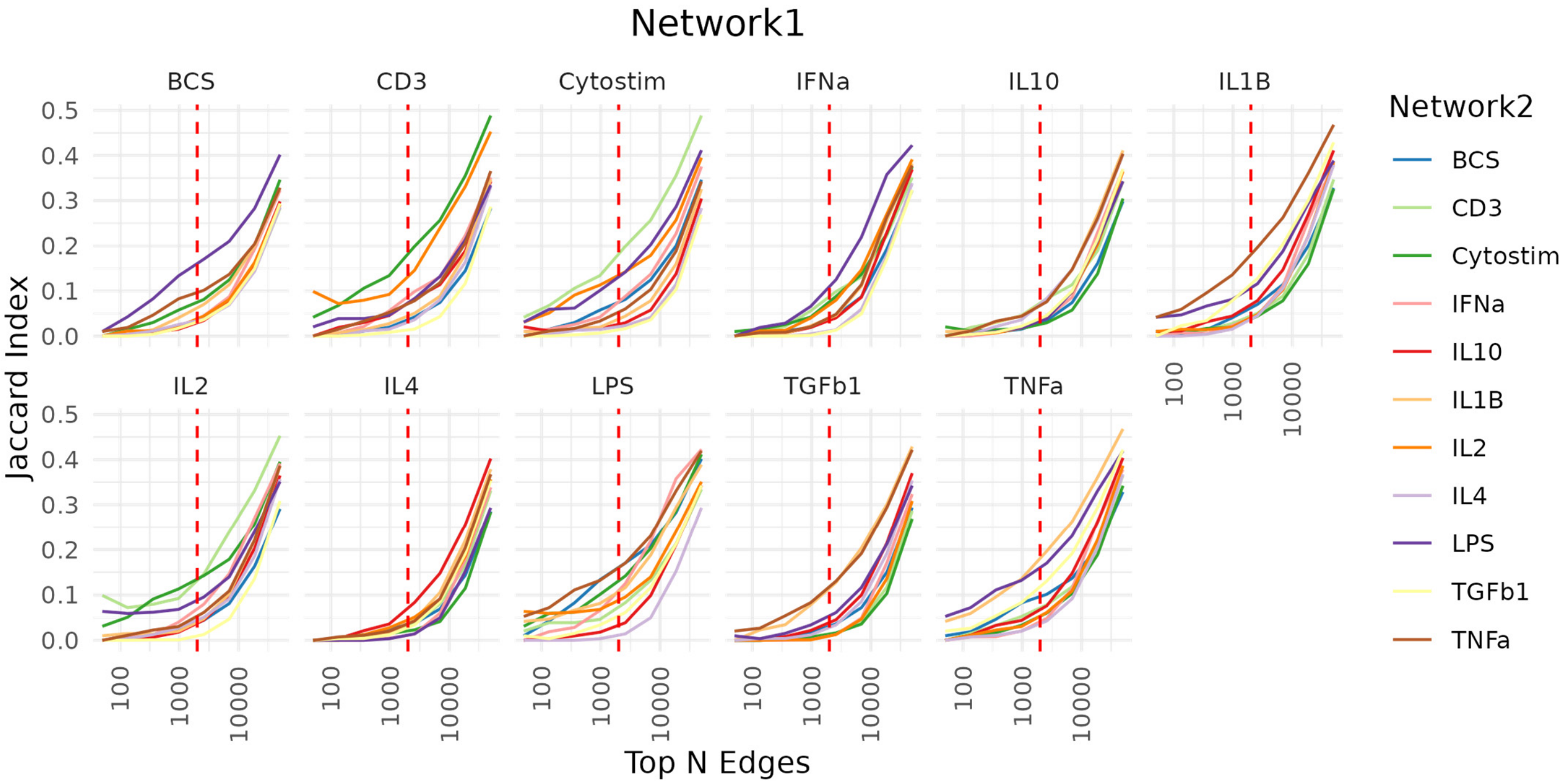


**Figure S4**

Pairwise comparisons by Jaccard index of networks generated by taking the top N ligand-receptor interactions (network edges) by prioritisation score. The threshold of top 2000 edges was selected to give maximal distinction between conditions and is highlighted on the plots.

**Figure S5**

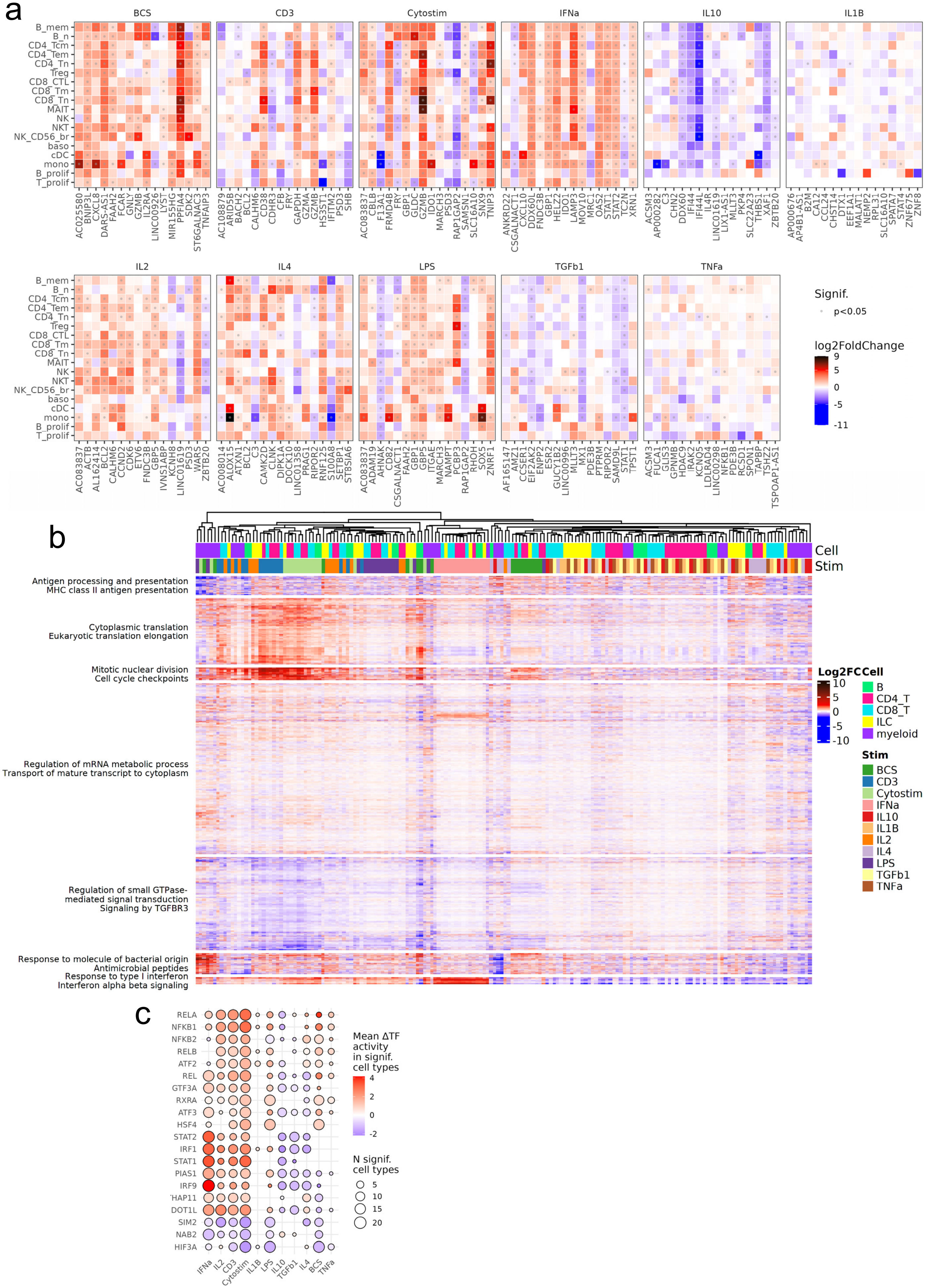
a
BCS
CD3
Cytostim
IFNa
IL10
IL1B
IL2
IL4
LPS
TGFb1
TNFa
B_mem
B_n
CD4_Tcm
CD4_Tem
CD4_Tn
Treg
CD8_CTL
CD8_Tm
CD8_Tn
MAIT
NK
NKT
NK_CD56_br
baso
cDC
mono
B_prolif
T_prolif
Signif.
p<0.05
log2FoldChange
9
5
0
-5
-11
b
Cell
Stim
Antigen processing and presentation
MHC class II antigen presentation
Cytoplasmic translation
Eukaryotic translation elongation
Mitotic nuclear division
Cell cycle checkpoints
Regulation of mRNA metabolic process
Transport of mature transcript to cytoplasm
Regulation of small GTPase-
mediated signal transduction
Signaling by TGFBR3
Response to molecule of bacterial origin
Antimicrobial peptides
Response to type I interferon
Interferon alpha beta signaling
Log2FC
10
5
0
-5
-10
Cell
B
CD4_T
CD8_T
ILC
myeloid
Stim
BCS
CD3
Cytostim
IFNa
IL10
IL1B
IL2
IL4
LPS
TGFb1
TNFa
c
RELA
NFKB1
NFKB2
RELB
ATF2
REL
GTF3A
RXRA
ATF3
HSF4
STAT2
IRF1
STAT1
PIAS1
IRF9
HAP11
DOT1L
SIM2
NAB2
HIF3A
IFNa
IL2
CD3
Cytostim
IL1B
LPS
IL10
TGFb1
IL4
BCS
TNFa
Mean ΔTF
activity
in signif.
cell types
4
2
0
-2
N signif.
cell types
5
10
15
20

**Figure S5**
(A) Log2-fold changes for top 4 differentially expressed genes (DEGs) per lineage (B, T, ILC, myeloid) by lowest adjusted p-values, for each stimulation vs. baseline. (B) Heatmap of all log2-fold changes vs. baseline for a set of 4,000 most variable genes across pseudo-bulk transcriptomic profiles. Each column represents a minor cell type/stim, annotated per general cell type. Rows (genes) were clustered with K-means clusters (k = 7) with curated over-represented pathways shown for each cluster (GO BP and Reactome annotations). (C) Top 20 TFs by frequency of inferred altered activity vs. baseline (adjusted $p<0.05$) across all cell type-stimulation combinations. Data are summarised per TF/stimulation indicating the number of cell types in which they are differentially active, and mean delta TF activity across those cell types.

**Figure S6**

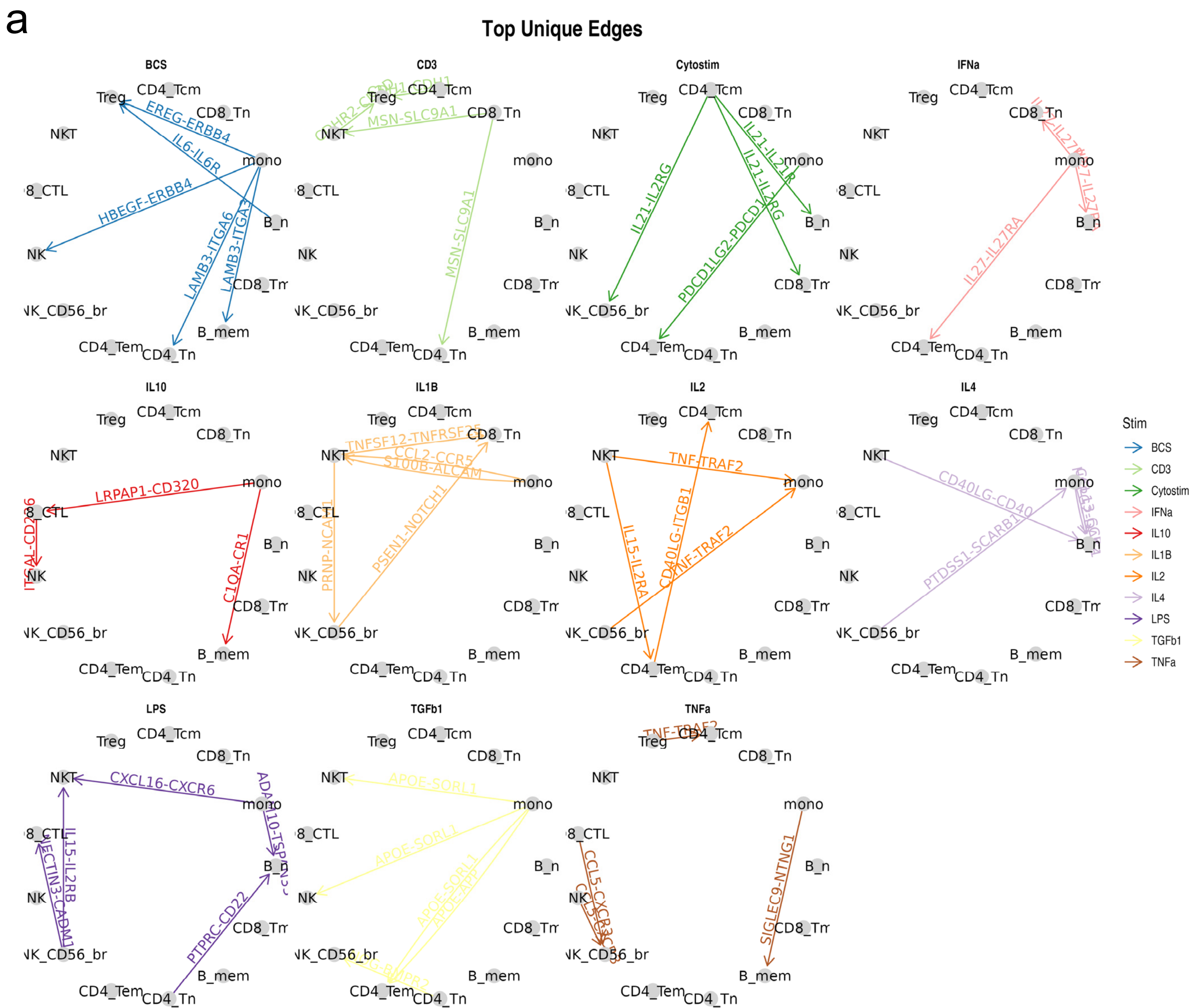
a
Top Unique Edges
BCS
CD3
Cytostim
IFNa
IL10
IL1B
IL2
IL4
LPS
TGFb1
TNFa
Stim
BCS
CD3
Cytostim
IFNa
IL10
IL1B
IL2
IL4
LPS
TGFb1
TNFa

# Figure S6

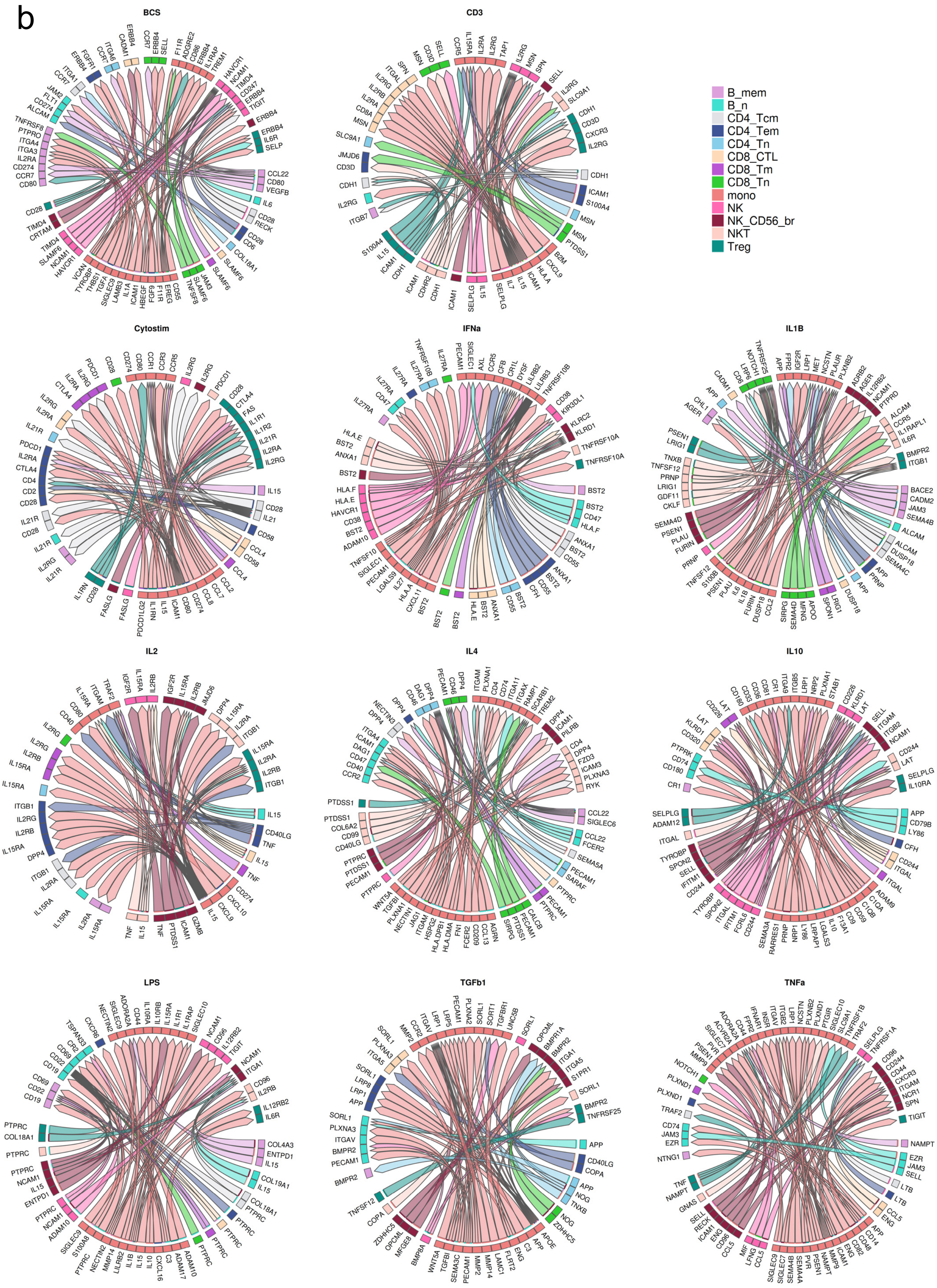
b
BCS
CD3
Cytostim
IFNa
IL1B
IL2
IL4
IL10
LPS
TGFb1
TNFa
B_mem
B_n
CD4_Tcm
CD4_Tem
CD4_Tn
CD8_CTL
CD8_Tm
CD8_Tn
mono
NK
NK_CD56_br
NKT
Treg

# Figure S6

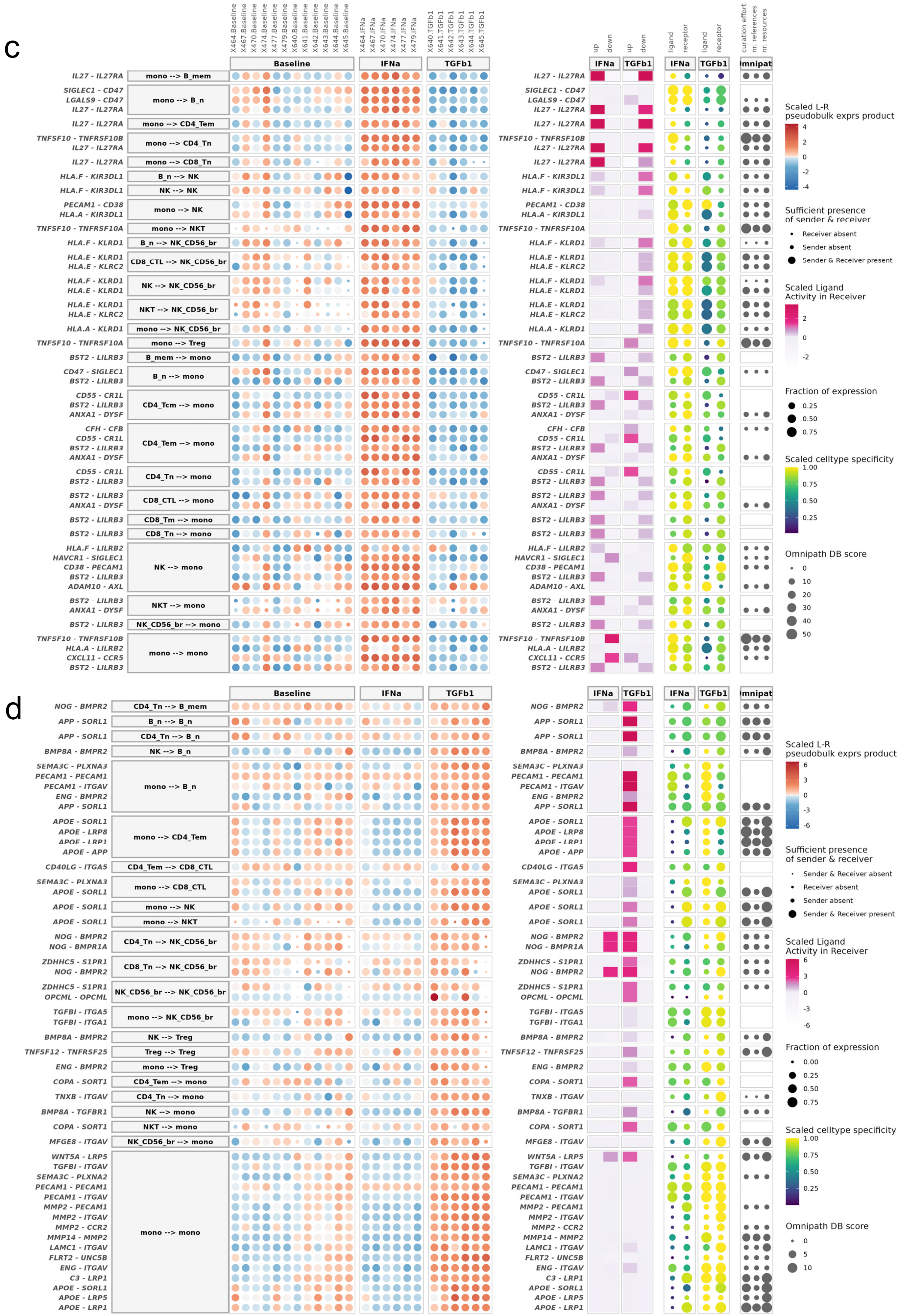

c
Baseline
IFNa
TGFb1
X464.Baseline
X467.Baseline
X470.Baseline
X474.Baseline
X477.Baseline
X479.Baseline
X640.Baseline
X641.Baseline
X642.Baseline
X643.Baseline
X644.Baseline
X645.Baseline
X464.IFNa
X467.IFNa
X470.IFNa
X474.IFNa
X477.IFNa
X479.IFNa
X640.TGFb1
X641.TGFb1
X642.TGFb1
X643.TGFb1
X644.TGFb1
X645.TGFb1
up
down
ligand
receptor
curation effort
nr. references
nr. resources
Omnipath
IL27 - IL27RA
mono --> B_mem
SIGLEC1 - CD47
LGALS9 - CD47
mono --> B_n
mono --> CD4_Tem
TNFSF10 - TNFRSF10B
mono --> CD4_Tn
mono --> CD8_Tn
HLA.F - KIR3DL1
B_n --> NK
NK --> NK
PECAM1 - CD38
HLA.A - KIR3DL1
mono --> NK
TNFSF10 - TNFRSF10A
mono --> NKT
HLA.F - KLRD1
B_n --> NK_CD56_br
HLA.E - KLRD1
HLA.E - KLRC2
CD8_CTL --> NK_CD56_br
NK --> NK_CD56_br
NKT --> NK_CD56_br
HLA.A - KLRD1
mono --> NK_CD56_br
mono --> Treg
BST2 - LILRB3
B_mem --> mono
CD47 - SIGLEC1
B_n --> mono
CD55 - CR1L
ANXA1 - DYSF
CD4_Tcm --> mono
CFH - CFB
CD4_Tem --> mono
CD4_Tn --> mono
CD8_CTL --> mono
CD8_Tm --> mono
CD8_Tn --> mono
HLA.F - LILRB2
HAVCR1 - SIGLEC1
CD38 - PECAM1
ADAM10 - AXL
NK --> mono
NKT --> mono
NK_CD56_br --> mono
HLA.A - LILRB2
CXCL11 - CCR5
mono --> mono
Scaled L-R pseudobulk exprs product
4
2
0
-2
-4
Sufficient presence of sender & receiver
Receiver absent
Sender absent
Sender & Receiver present
Scaled Ligand Activity in Receiver
2
0
-2
Fraction of expression
0.25
0.50
0.75
Scaled celltype specificity
1.00
0.75
0.50
0.25
Omnipath DB score
0
10
20
30
40
50
d
NOG - BMPR2
CD4_Tn --> B_mem
APP - SORL1
B_n --> B_n
CD4_Tn --> B_n
BMP8A - BMPR2
NK --> B_n
SEMA3C - PLXNA3
PECAM1 - PECAM1
PECAM1 - ITGAV
ENG - BMPR2
mono --> B_n
APOE - SORL1
APOE - LRP8
APOE - LRP1
APOE - APP
mono --> CD4_Tem
CD40LG - ITGA5
CD4_Tem --> CD8_CTL
mono --> CD8_CTL
mono --> NK
mono --> NKT
NOG - BMPR1A
CD4_Tn --> NK_CD56_br
ZDHHC5 - S1PR1
CD8_Tn --> NK_CD56_br
OPCML - OPCML
NK_CD56_br --> NK_CD56_br
TGFBI - ITGA5
TGFBI - ITGA1
mono --> NK_CD56_br
NK --> Treg
TNFSF12 - TNFRSF25
Treg --> Treg
mono --> Treg
COPA - SORT1
CD4_Tem --> mono
TNXB - ITGAV
CD4_Tn --> mono
BMP8A - TGFBR1
NK --> mono
NKT --> mono
MFGE8 - ITGAV
NK_CD56_br --> mono
WNT5A - LRP5
TGFBI - ITGAV
SEMA3C - PLXNA2
MMP2 - PECAM1
MMP2 - ITGAV
MMP2 - CCR2
MMP14 - MMP2
LAMC1 - ITGAV
FLRT2 - UNC5B
ENG - ITGAV
C3 - LRP1
APOE - LRP5
mono --> mono
Scaled L-R pseudobulk exprs product
6
3
0
-3
-6
Sufficient presence of sender & receiver
Sender & Receiver absent
Receiver absent
Sender absent
Sender & Receiver present
Scaled Ligand Activity in Receiver
6
3
0
-3
-6
Fraction of expression
0.00
0.25
0.50
0.75
Scaled celltype specificity
1.00
0.75
0.50
0.25
Omnipath DB score
0
5
10

**Figure S6**

Upregulated ligand-receptor interactions were predicted per condition compared to baseline with MultiNicheNet. (A) Up to 5 top unique ligand-receptor network edges are shown per condition.. (B) Top 50 interactions per condition vs. baseline. Arrows indicate direction of signalling from sender to receiver cell type. (C) and (D) highlight the supporting data for the top 50 upregulated ligand-receptor interactions in the IFN-α and TGF-β1 conditions, respectively, compared to baseline. Data shown per ligand-receptor and sender-receiver cell (1 per row), from left to right are: scaled ligand-receptor pseudo-bulk expression product and sender/receiver cell presence per donor/condition; downstream ligand activity (up- or down-regulated target genes) in receiver cells; cell type-specificity and fraction of cells expressing ligand/receptor; summary of evidence for interaction (Omnipath).

# Figure S7

a

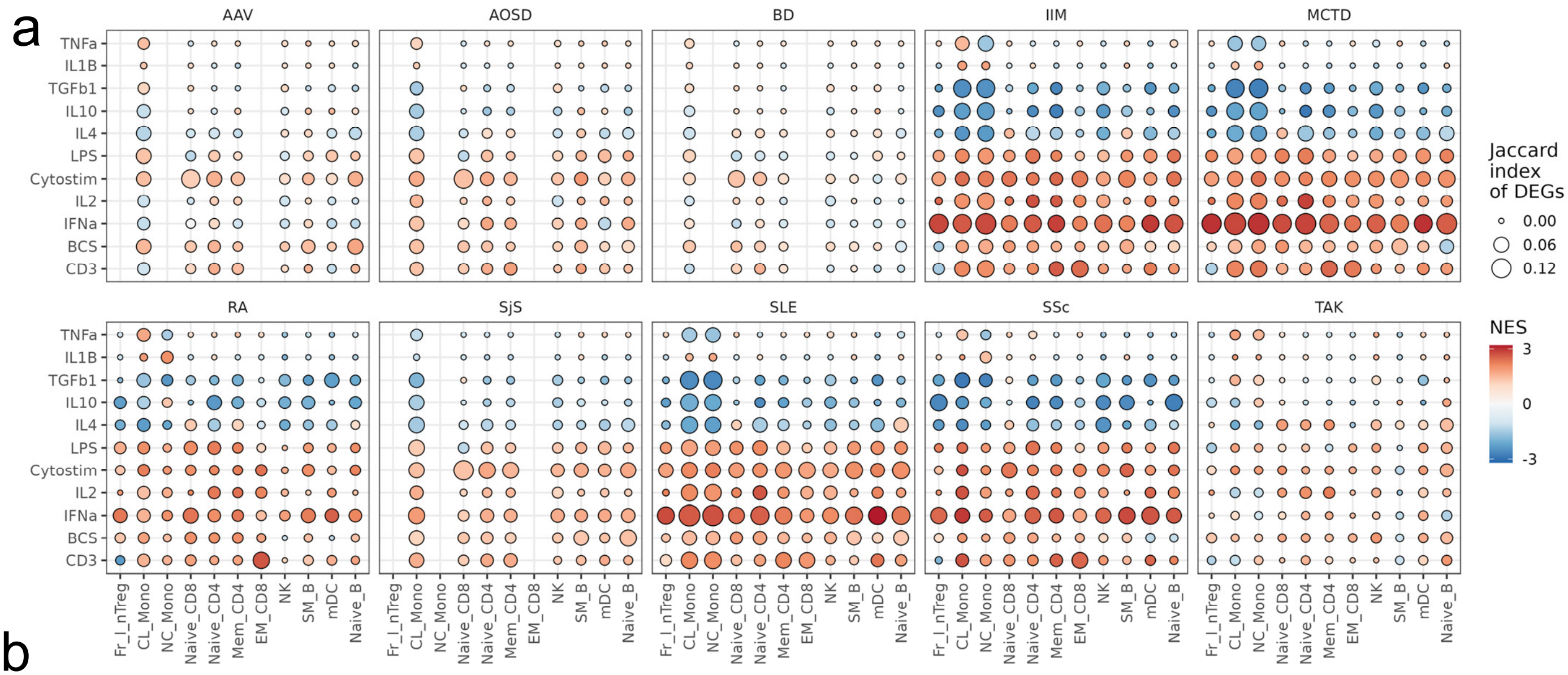


b

| Major lineage | Cell subset | Core definition (simplified) |
|---|---|---|
| **CD4 T cells** | Naïve CD4 T cells | CD3$^{+}$ CD4$^{+}$ CD45RA$^{+}$ CCR7$^{+}$ |
| | Memory CD4 T cells | CD3$^{+}$ CD4$^{+}$ CD45RA$^{-}$ |
| | T helper 1 cells | CXCR3$^{+}$ CCR6$^{-}$ |
| | T helper 2 cells | CCR4$^{+}$ CXCR3$^{-}$ |
| | T helper 17 cells | CCR6$^{+}$ CXCR3$^{-}$ |
| | T follicular helper cells | CXCR5$^{+}$ |
| | Regulatory T cells (naïve) | CD25$^{+}$ CD45RA$^{+}$ |
| | Regulatory T cells (effector) | CD25$^{++}$ CD45RA$^{-}$ |
| | Non-regulatory CD4 T cells | CD25$^{+}$ CD45RA$^{-}$ (non-Treg) |
| **CD8 T cells** | Naïve CD8 T cells | CD8$^{+}$ CD45RA$^{+}$ CCR7$^{+}$ |
| | Central memory CD8 T cells | CD8$^{+}$ CD45RA$^{-}$ CCR7$^{+}$ |
| | Effector memory CD8 T cells | CD8$^{+}$ CD45RA$^{-}$ CCR7$^{-}$ |
| | TEMRA CD8 T cells | CD8$^{+}$ CD45RA$^{+}$ CCR7$^{-}$ |
| **B cells** | Naïve B cells | IgD$^{+}$ CD27$^{-}$ |
| | Unswitched memory B cells | IgD$^{+}$ CD27$^{+}$ |
| | Switched memory B cells | IgD$^{-}$ CD27$^{+}$ |
| | Double-negative B cells | IgD$^{-}$ CD27$^{-}$ |
| | Plasmablasts | CD27$^{++}$ CD38$^{+}$ |
| **Natural killer cells** | NK cells | CD3$^{-}$ CD56$^{+}$ |
| **Monocytes** | Classical monocytes | CD14$^{++}$ CD16$^{-}$ |
| | Intermediate monocytes | CD14$^{+}$ CD16$^{+}$ |
| | Non-classical monocytes | CD14$^{+dim}$ CD16$^{+}$ |
| **Dendritic cells** | Myeloid dendritic cells | CD11c$^{+}$ CD123$^{-}$ |
| | Plasmacytoid dendritic cells | CD11c$^{-}$ CD123$^{+}$ |
| **Granulocytes** | Low-density granulocytes | SSC^high CD15$^{+}$ CD16$^{+}$ |
| | Neutrophils | Density-isolated neutrophils |

| Diagnosis | N | Age (years) | Av. Disease duration (years) |
|---|---|---|---|
| Healthy control | 79 | 48 (38–58) | NA |
| ANCA-associated vasculitis (AAV) | 25 | 68 (59–76) | 6 (4.3–12) |
| Adult-onset Still's disease (AOSD) | 18 | 47 (34–59) | 6 (3.2–12) |
| Behçet's disease (BD) | 23 | 54 (50–62) | 15 (10–22) |
| Mixed connective tissue disease (MCTD) | 19 | 49 (42–64) | 17 (9.5–21) |
| Idiopathic inflammatory myopathy (IIM) | 65 | 57 (46–69) | 7 (0–12) |
| Rheumatoid arthritis (RA) | 24 | 63 (54–70) | 5 (0.8–20) |
| Sjögren's syndrome (SjS) | 18 | 59 (52–68) | 12 (1.2–22) |
| Systemic lupus erythematosus (SLE) | 62 | 47 (39–58) | 12 (4–21) |
| Systemic sclerosis (SSc) | 67 | 67 (58–74) | 15 (7–26) |
| Takayasu arteritis (TAK) | 16 | 58 (39–74) | 24 (11–34) |

Immune cell subsets and patients profiled in the ImmuNexUT cohort

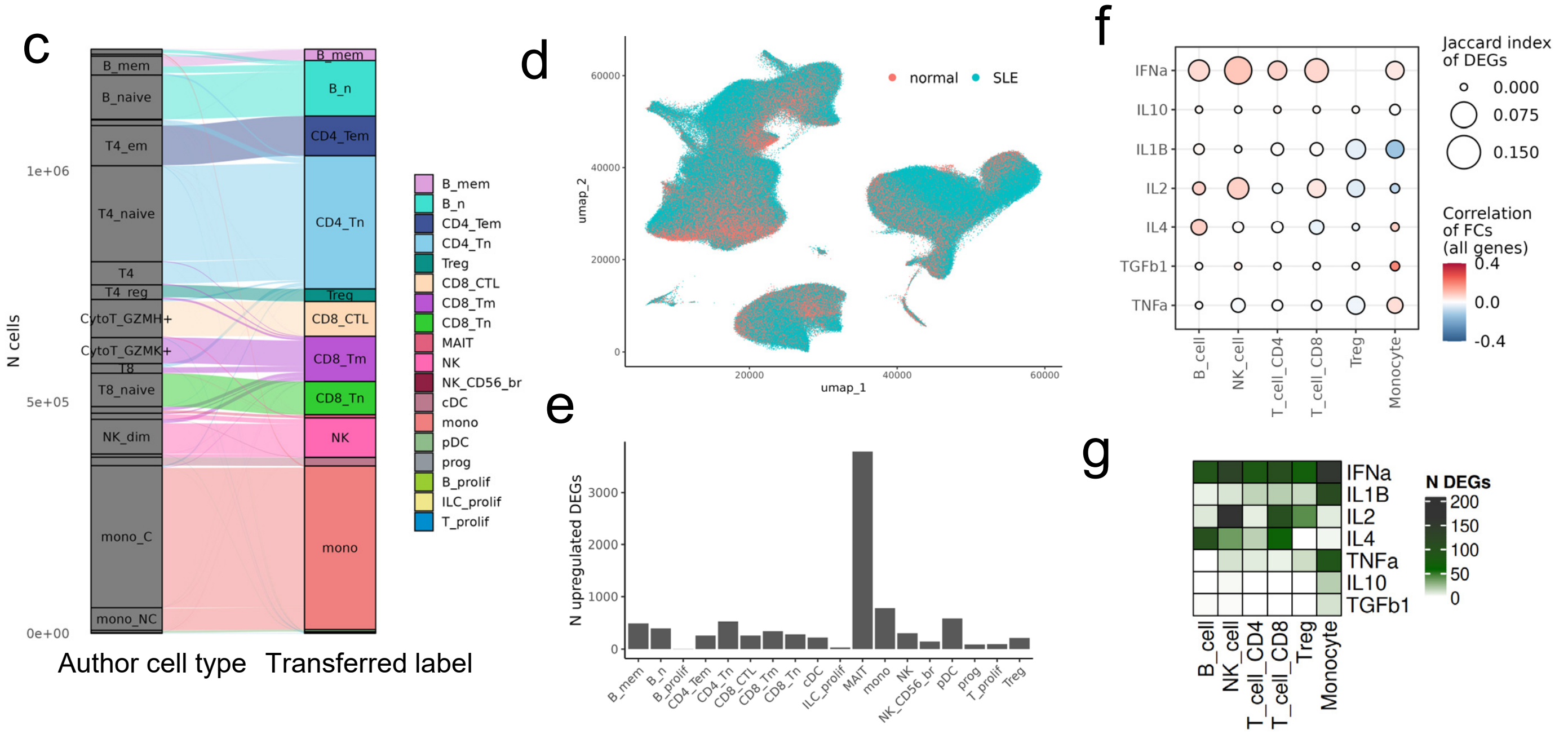

**Figure S7**

(A) Gene set enrichment of cell type-specific disease gene sets (defined as adjusted p<0.01, log2-fold change >0.5; disease vs. healthy) from bulk sorted PBMCs (ImmunexUT dataset -systemic lupus erythematosus (SLE), idiopathic inflammatory myopathy (IIM), systemic sclerosis (SSc), mixed connective tissue disease (MCTD), Sjögren’s syndrome (SjS), rheumatoid arthritis (RA), Behçet's disease (BD), adult-onset Still's disease (AOSD), ANCA-associated vasculitis (AAV), or Takayasu arteritis (TAK) ) within ranked gene profiles (ranked by Wald stat; logFC/lfcSE; stimulation vs. baseline) of matching cell types of stimulated PBMC profiles. Enrichment is summarised as normalised enrichment score (NES; shown by colour). Overlap of significant gene sets between disease and stimulation (defined as adjusted p<0.01, log2-fold change >0.5; stimulated vs. baseline) was also assessed by Jaccard index (shown by size). Missing points indicate missing cell type/condition within the ImmunexUT dataset. (B) Summary of immune cell subsets and patients profiled in the ImmuNexUT cohort. (C) Reannotation of SLE and normal cells from a single-cell RNA-seq dataset to match cell type labels. (D) UMAP dimensional reduction showing the clustering of SLE and normal cells. (E) Number of upregulated differentially expressed genes (DEGs) per cell type in pseudo bulk profiles of SLE cells vs. healthy control. (F) Comparison of transcriptomic profiles from *in vivo* mouse cytokine treatment (Immune Dictionary dataset) vs. *in vitro* human PBMC stimulations, for equivalent cell type annotations and conditions used in both datasets. Size indicates Jaccard index of DEGs per cell type/condition, defined as those with adjusted p<0.05. Colour indicates Spearman correlation of log2-fold changes for all genes with direct or orthologous identifiers between human and mouse. (G) Summary of the number of DEGs per cell type/condition from *in vivo* mouse cytokine treatment.
